# A novel rat model of vertebral inflammation–induced intervertebral disc degeneration mediated by activating cGAS/STING molecular pathway

**DOI:** 10.1111/jcmm.16898

**Published:** 2021-09-03

**Authors:** Qihang Su, Qiuchen Cai, Yongchao Li, Hengan Ge, Yuanzhen Zhang, Yi Zhang, Jun Tan, Jie Li, Biao Cheng, Yan Zhang

**Affiliations:** ^1^ Department of Orthopedics, Shanghai Tenth People's Hospital School of Medicine, Tongji University Shanghai China; ^2^ Department of Orthopedics, Shanghai East Hospital School of Medicine, Tongji University Shanghai China; ^3^ Department of Orthopedics Shanghai Zhabei District Central Hospital Shanghai China; ^4^ Department of Bone and Soft Tissue Tumors Tianjin Medical University Cancer Institute and Hospital Tianjin China

**Keywords:** animal model, cGAS/STING signalling pathway, intervertebral disc degeneration, spread of inflammation, vertebral inflammation–induced intervertebral disc degeneration model

## Abstract

In this study, we describe a new rat model of vertebral inflammation–induced caudal intervertebral disc degeneration (VI‐IVDD), in which IVD structure was not damaged and controllable segment and speed degeneration was achieved. VI‐IVDD model was obtained by placing lipopolysaccharide (LPS) in the caudal vertebral bodies of rats. Rat experimental groups were set as follows: normal control group, group with a hole drilled in the middle of vertebral body and not filled with LPS (Blank group), group with a hole drilled in the middle of vertebral body and filled with LPS (Mid group), and group with hole drilled in the vertebral body in proximity of IVD and filled with LPS (NIVD group). Radiological results of VI‐IVDD rats showed a significant reduction in the intervertebral space height and decrease in MRI T2 signal intensity. Histological stainings also revealed that the more the nucleus pulposus and endplate degenerated, the more the annulus fibrosus structure appeared disorganized. Immunohistochemistry analysis demonstrated that the expression of Aggrecan and collagen‐II decreased, whereas that of MMP‐3 increased in Mid and NIVD groups. Abundant local production of pro‐inflammatory cytokines was detected together with increased infiltration of M1 macrophages in Mid and NIVD groups. Apoptosis ratio remarkably enhanced in Mid and NIVD groups. Interestingly, we found a strong activation of the cyclic GMP‐AMP synthase /stimulator of interferon gene signalling pathway, which is strictly related to inflammatory and degenerative diseases. In this study, we generated a new, reliable and reproducible IVDD rat model, in which controllable segment and speed degeneration was achieved.

## 1 INTRODUCTION

About 18%–48% of the population will experience chronic low back pain (LBP) at some time during life, and most LBP patients show intervertebral disc degeneration (IVDD).^1^, ^2^ IVDD is a complex age‐related process^3^ with a multifaceted aetiology including signs such as inflammation,^4^ micro‐damage,^5^ biomechanical stress,^6^ ageing,^7^ apoptosis^8^ and autophagy.^9^ However, the integrated molecular mechanisms underlying IVDD have not been fully elucidated,^10^ and no effective and reliable intervertebral disc (IVD) repair strategy has been developed so far.^11^ As a result, symptomatic or alternative treatments are often adopted for IVDD‐induced spinal diseases in clinic. Hence, much efforts are needed to identify the mechanisms underlying IVDD and potential therapeutical targets, and the generation of highly reliable and reproducible animal models of IVDD will be fundamental.

Due to factors, such as animal ethics, source, costs, human similarity and ease of manipulation, the most commonly used animals for establishing IVDD models are rats/mice and rabbits.^12^ IVDD animal models are mainly divided into two categories: induced IVDD model (abnormal stress model,^13^ spinal instability model,^14^ mechanical damage model,^15^ chemical induction model^16^ and biological induction model^17^) and spontaneous IVDD model (endocrine change model^18^ and standing model^19^).

Attempts have been made to induce IVDD by changing the IVD stress in many studies. Lai et al.^13^ induced caudal IVDD in rats through abnormal axial stress caused by pressurizing the rat tail. Our previous studies showed that IVDD could also be obtained by applying shear force to rabbit IVDs.^20^ Meanwhile, Miyamoto et al.^14^ generated IVDD by surgically inducing the instability of the spine. The degeneration model dependent by stress change strongly resembles IVDD, since it simulates the effects of stress change caused by different postures. The standing model of spontaneous IVDD is similar to these models. Ao et al.^19^ developed an animal model in which IVDD was induced by continuously stimulating the mice to take bipedal standing posture, perfectly simulating the process of natural IVDD induced by human standing. However, the long‐time duration [(1 h/day) × 1 month to (6 h/day) × 2 months) and low success rate limit research on IVDD condition in these models.

To simulate the process of natural IVDD in humans, gene editing or ovariectomy^17^, ^18^ has also been used to induce IVDD in animals. However, the high costs, as well as the scarce experimental reproducibility and reliability, represent a limit of these methods. Hence, there is a need to establish easy‐to‐handle models, with low costs and high success rate, such as those induced by chemicals and mechanical damage. Chemical induction models are obtained by injection of protease, collagenase or bleomycin into the IVD.^16^, ^21^ The most typical mechanical damage model is the rat caudal IVD acupuncture model, which is currently also the most widely applied model.^8^, ^15^ However, irreversible damage to IVD structure is main limitation of these models. In fact, according to previous and our observations,^8^, ^22^ once the IVD structure is damaged, degeneration rapidly and irreversibly progresses. Animal models in which IVD structure is compromised cannot simulate natural IVDD human condition, such as age‐related IVDD.

A large number of studies^8^, ^15^, ^23^ have shown that IVDD models induced by mechanical damage are highly reproducible and dependent on local inflammatory responses induced by IVD damage. Although inflammation is an important factor for rapid induction of IVDD,^4^ IVD structure damage obtained by mechanical stress in animals is unsuitable for studying natural degenerative process.

**FIGURE 1:**
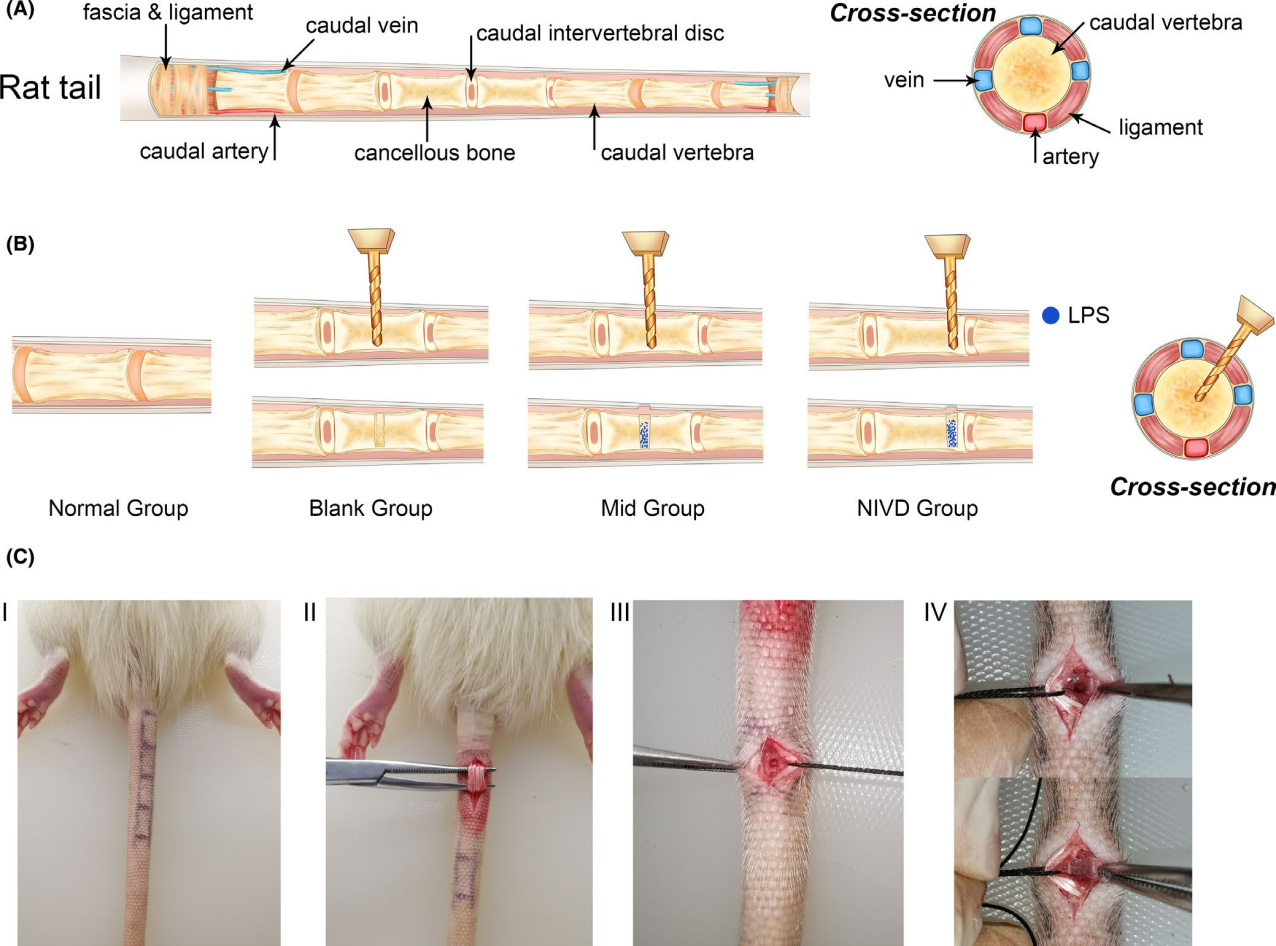
Intervertebral disc degenerationmodel (IVDD) establishment. (A) Anatomy of rat tail; (B) experimental groups included the normal control group (Normal group), group with hole drilled in the middle of vertebral body without placing lipopolysaccharide (LPS) (Blank group), group with hole drilled in the middle of vertebral body and treated with LPS (Mid group, also called VI‐IVDD basic model), and group with hole drilled at one side (in proximity of IVD) of the vertebral body and treated with LPS (NIVD group, also called VI‐IVDD acceleration model); (C) experimental procedure included the following steps: (I) localization of vertebral body, IVD and vessel; (II) dissection and localization of subcutaneous tissue and caudal ligament; (III) identification of drilling position, and execution of drilled holes in vertebral bodies with a drill bit (diameter: 1.5 mm), inserted with a 45‐degree inclination; and (IV) in Mid and NIVD groups, LPS was placed and sealed with bone wax, whereas in the Blank group, drilled hole was sealed with bone wax without LPS. After surgical intervention, skin was sutured and disinfected

In this study, we describe a new, highly reliable and reproducible IVDD rat model obtained by inducing inflammation in the vertebral body without affecting the integrity of IVD structure. In particular, IVDD was induced by placing the pro‐inflammatory agent lipopolysaccharide (LPS) in the middle of the caudal vertebral bodies of rats. LPS treatment determined the triggering of rapid, stable and localized inflammatory responses,^24^ with concomitant degeneration of the adjacent IVD. In this vertebral inflammation–induced IVDD (VI‐IVDD) model (Figure 1), the progression of inflammation was studied by using radiological imaging, as well as by performing morphological, histological and cytological evaluations. The involvement of LPS‐induced cyclic GMP‐AMP synthase (cGAS)/stimulator of interferon genes (STING) signalling pathway^25^ was also investigated in the model.

## 2 MATERIALS AND METHODS

### 2.1 Animals

Ten to 12‐week‐old male SPF‐grade Sprague Dawley rats (Shanghai SLAC Laboratory Animal Co., Ltd.; *n* = 192), with an average bodyweight of 250–300 g, were randomly divided into four groups (*n* = 48 per group): normal control group (Normal group), group with a hole drilled in the middle of vertebral body and not filled with LPS (Blank group), group with a hole drilled in the middle of vertebral body and filled with LPS (Mid group, also called VI‐IVDD basic model), and group with hole drilled in the vertebral body in the proximity of IVD and filled with LPS (in the proximity of intervertebral disc, NIVD group, also called VI‐IVDD acceleration model). The progression of inflammation and degeneration were studied at 1, 2 and 4 weeks after surgical intervention, with 16 rats being randomly selected for analysis for each group and time‐point.

No accidental death or operation failure occurred in rats used for the study. Skin surgical incision in rats healed well, and no infections were observed at cutaneous or subcutaneous soft tissue levels.

At the end of experimental procedure, rats were anaesthetized and killed by cervical dislocation.

All surgical interventions, treatments and animal care procedures were performed in strict accordance with the Animal Care and Use Committee of the University School of Medicine.

### 2.2 Model establishment

Rats underwent intraperitoneal injection of 100 mg/kg ketamine + 8 mg/kg xylazine (Sangon Biotech),^26^ and after being deeply anaesthetized, surgical interventions were made.

Caudal vertebral bodies, IVDs and large vessels have been previously localized in the caudal region of rats (Figure 1A). In particular, the 9th or 10th caudal vertebral bodies were localized by touching the ilium and sacrum of the rats, and the area where the hair disappeared at the junction of trunk and tail (in proximity of the 9th caudal vertebral body). Four large subcutaneous vessels in the tail of rats, visible to naked eyes, have also been localized in the dorsal and ventral midlines, and in both sides from the middle of the tail.

Normal group rats were not treated, whereas the other three groups were subjected to hole drilling into vertebral bodies (Figure 1B). For drilling, it was necessary to make a longitudinal incision (0.5 cm) in the skin, carefully avoiding vessels. The subcutaneous fascia and ligament were separated, and the modelling vertebral body was exposed (Figure 1CI,II). In Blank and Mid groups, a drill bit (diameter: 1.5 mm) was drilled into the middle of the vertebral body, with an inclination of 45°, without going through the vertebral body. In the NIVD group, the hole has been drilled into the vertebral body in the proximity of one side of IVD (about 2–4 mm; Figure 1B,C‐III). After drilling, 0.25 mg of LPS powder (*Escherichia coli* O111:B4, Sigma‐Aldrich Co., Ltd.) was placed into the drilled hole in rats of Mid and NIVD groups and sealed with bone wax (Figure 1C‐IV) in order to prevent LPS leakage out of the vertebral body and infections of the subcutaneous soft tissue. Based on previous team experience, IL‐1β, IL‐6 and TNF‐α cytokines were not used as inflammatory triggers in the model, as they are high‐cost, easily degradable, not capable to sustain local inflammatory responses and quite often responsible for systemic inflammation. Skin was then sutured and disinfected. When the rats were fully awake, they were moved to the animal room and treated normally. The incision skin was daily disinfected, and the local pain was relieved with a mixture solution of 75% alcohol and 20 g/ml lidocaine (1:1) to prevent skin infections and impact on experiments.

### 2.3 Radiological evaluation

Caudal IVD and intervertebral space of rats of different groups were radiologically evaluated at the different time‐points. According to our previous research, molybdenum‐target plain X‐ray image is superior to plain X‐ray in assessing intervertebral space in rats.^22^ Hence, molybdenum‐target plain X‐ray system (Mammomat Inspiration, Siemens) was adopted to obtain images of vertebral bodies and intervertebral space. Disc height index (DHI) was measured to evaluate the change in intervertebral space height and calculated as follows: DHI = 2 × (D1 + D2 + D3)/[(PV1 + PV2 + PV3) + (DV1 + DV2 + DV3)] (Figure S1).^8^, ^22^, ^27^ Magnetic resonance imaging (MRI) (uMR770; Shanghai United Imaging Intelligence Healthcare Co., Ltd.) was performed on the tail of rats, and Adobe Photoshop software (CC 2018, Adobe Systems Incorporated) was used to measure the total greyscale value of IVD adjacent to the operating vertebral body (a smaller value indicated severer degeneration),^28^ and thus to quantitatively analyse the severity of IVDD.

### 2.4 Sampling

The operated vertebral body, the adjacent IVD and a half of the adjacent vertebral body were kept and fixed with 4% paraformaldehyde. Samples were then decalcified, dehydrated and embedded in paraffin. Four‐micrometer sections were deparaffinized and preserved on a glass slide at room temperature (pathology slicer and Leica embedder provided by Shanghai Leica Instrument Co., Ltd.) until stainings were performed.

### 2.5 Histological staining

Sections were subjected to haematoxylin‐eosin (HE), safranin O/fast green (SOFG) and Masson's trichrome histological stainings, according to conventional procedures^8^, ^29^ and using standard HE, SOFG and Masson dye solutions (Wuhan Servicebio Technology Co., Ltd.). Microscope images were kept at different magnifications, using a scanning imaging system (ECLIPSE E100 and DS‐U3, NIKON).

### 2.6 Immunohistochemistry analysis

Immunohistochemical (IHC) indexes of Aggrecan, collagen‐II (Col‐II), matrix metalloproteinase‐3 (MMP‐3), IL‐1β, IL‐6, TNF‐α and interferon regulatory factor 3 (IRF3) were evaluated. To this end, sections were subjected to IHC according to conventional procedures,^8^ as follows: (1) deparaffinizing and rehydrating the paraffin section; (2) antigen retrieval; (3) blocking endogenous peroxidase activity; (4) serum sealing; (5) primary antibody incubation (primary antibody/phosphate buffer saline = 1/100, Servicebio); (6) secondary antibody incubation and DAB chromogenic reaction; (7) nucleus counterstaining; (8) dehydration and mounting; and (9) obtaining staining microscope images at different magnifications and acquisition at imaging system. Quantifications of stainings were performed by measuring the average optical density (AOD) of each index using the Image‐Pro Plus 6.0 software.

### 2.7 Fluorescein isothiocyanate‐TdT‐mediated dUTP nick‐end labelling assay

TdT‐mediated dUTP nick‐end labelling (TUNEL) assay was used to evaluate cell apoptosis.^30^ Paraffin sections were deparaffinized and rehydrated and then treated with proteinase K to permeabilize cell membranes. Next, TUNEL kit reaction solution (TDT enzyme:fluorescein‐dUTP:buffer = 1:5:50) was added to sections, which were left in a constant temperature incubator at 37°C for 2 h. Nuclei were counterstained with 4',6‐diamidino‐2‐phenylindole (DAPI), and the sections were mounted. Images were kept using a fluorescence microscope (DAPI UV excitation wavelength: 330–380 nm, emission wavelength: 420 nm, blue light; fluorescein isothiocyanate (FITC) excitation wavelength: 465–495 nm, emission wavelength: 515–555 nm, green light). Finally, the positive rate of apoptotic cells was calculated.

### 2.8 Immunofluorescence

M1 macrophage markers, such as inducible nitric oxide synthase (iNOS) and CD68,^31^, ^32^ cGAS and STING (endoplasmic reticulum protein) proteins of the cGAS/STING signalling pathway, and TANK‐binding kinase 1 (TBK1),^25^, ^33^ were detected by immunofluorescence (IF). Double‐IF labelling was adopted for iNOS, CD68, cGAS and STING.^34^

After quenching endogenous peroxidase with 3% H_2_O_2_, achieving antigen retrieval and blocking nonspecific binding sites by serum, sections were incubated at RT with primary (macrophage: iNOS, signalling pathway: cGAS) and then secondary antibodies. FITC‐TSA was added to develop and amplify IF signals. Primary and secondary antibodies bound to the tissue were removed using microwave. The second immunostaining was performed with primary antibodies (macrophages: CD68, signalling pathway: STING) and the corresponding secondary antibody (Cy3). DAPI was used for counterstaining of nuclei. Finally, after the autofluorescence was quenched, the sections were mounted, examined at microscope, and photographed (DAPI UV excitation wavelength: 330–380 nm, emission wavelength: 420 nm, blue light; FITC excitation wavelength: 465–495 nm, emission wavelength: 515–555 nm, green light; Cy3 excitation wavelength: 510–560, emission wavelength: 590 nm, red light). For TBK1, after adding primary and secondary antibody (Cy5) in accordance with the above procedures, DAPI counterstaining and subsequent steps were carried out. The antibodies were all provided by ABclonal Co., Ltd. AOD of each marker was measured using the Image‐Pro Plus 6.0 software.

### 2.9 Statistical analysis

All data were analysed using IBM SPSS Statistics for Windows, version 20 (IBM Corp.). *p* < 0.05 was considered statistically significant. According to data type (obvious interaction between groups and time variables), simple effect analysis of factorial design was adopted to analyse the DHI of molybdenum‐target X‐ray images (interaction: *F* = 14.564, *p* < 0.05; Table 1), the total greyscale value of IVD in MRI (interaction: *F* = 26.000, *p* < 0.05; Table 1), AOD data of Aggrecan, Col‐II, MMP‐3, IL‐1β, IL‐6 and TNF‐α, and the positive rate of apoptotic cells. A nonparametric Kruskal‐Wallis (K‐W) test was used to analyse the positive rate of macrophages and AOD data of cGAS, STING, TBK1 and IRF3 since the data were not in line with normal distribution and homogeneity of variance. Pearson's correlation coefficient and overlap coefficient were used to analyse data of co‐localization between the macrophage markers iNOS and CD68, and cGAS and STING in the signalling pathway.

**TABLE 1:** Analysis of variance of factorial design in DHI and MRI

Variables		*F*	*p*
DHI	Group	166.525	0.000
	Time	23.433	0.000
	Group^b^ Time	14.564	0.000
MRI^a^	Group	315.492	0.000
	Time	63.037	0.000
	Group^b^ Time	26.000	0.000

Abbreviation: DHI, The disc height index.

^a^The grey value of degenerative discs in MRI.

^b^Interaction between Group and Time. Group: Normal, Blank, Mid, NIVD; Time: 1 week, 2 weeks, 4 weeks.

## 3 RESULTS

### 3.1 Radiological evaluation

**FIGURE 2:**
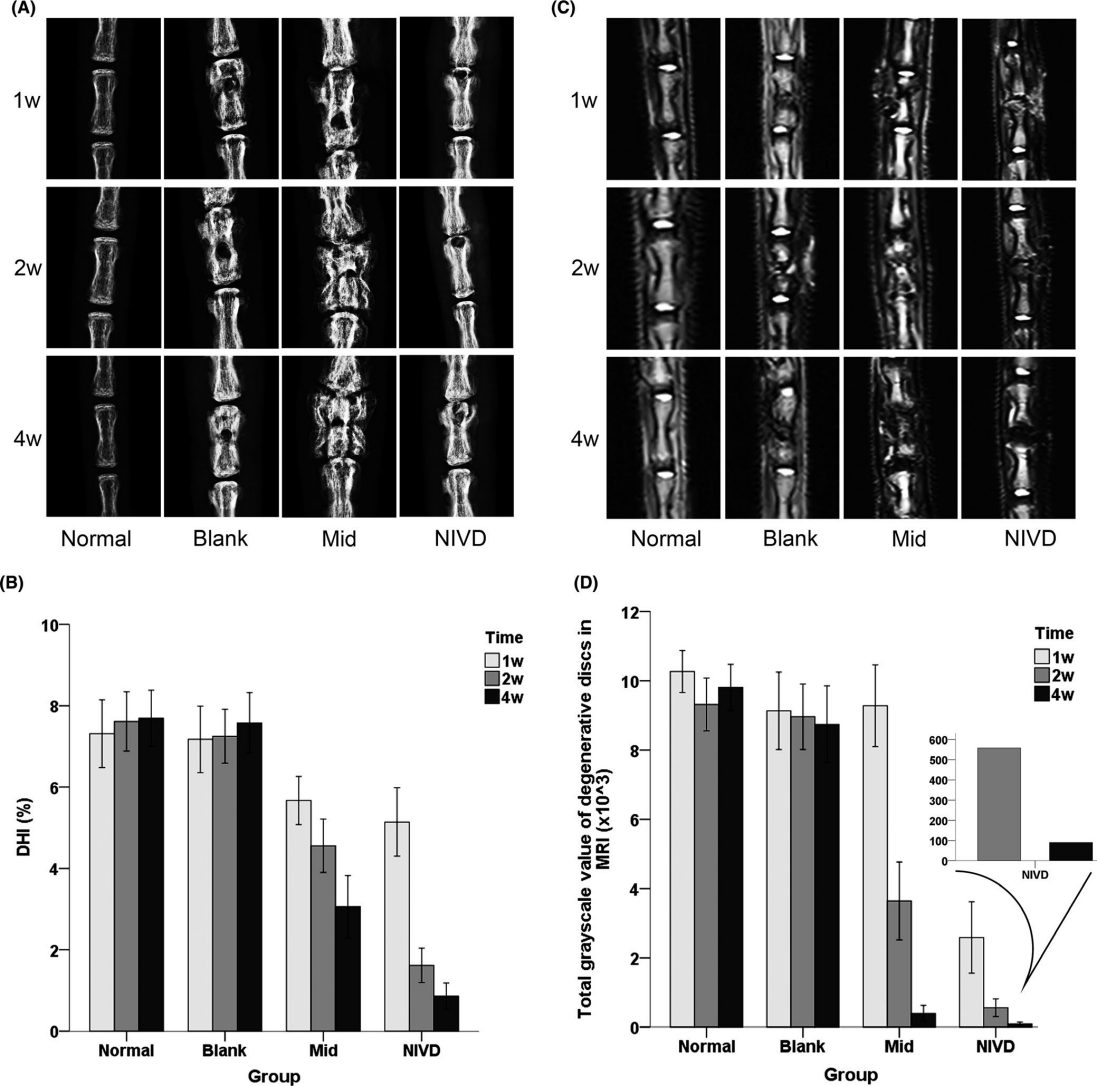
Radiological evaluation. (A) Intervertebral space height and the position of drilled holes were evaluated by molybdenum‐target plain X‐ray image at 1, 2 and 4 weeks for each group. The intervertebral space height decreased in Mid and NIVD groups; (B) statistical analysis relative to the quantitative index of intervertebral space height (DHI); (C) MRI images at 1, 2 and 4 weeks for each group. Preliminary evaluation of IVDD revealed that the T2 signal of IVD decreased in Mid and NIVD groups; (D) statistical analysis relative to the total greyscale value of MRI T2 signal of IVD

Vertebral body, adjacent intervertebral space and the drilling position were firstly visualized by molybdenum‐target plain X‐ray system in rats of the four experimental groups (Figure 2A). X‐ray images showed that holes were drilled accurately in Blank, Mid and NIVD groups and there was no direct damage to the IVD. Normal and Blank groups had a normal intervertebral space height, and no evident changes from 1 to 4 weeks were observed. Mid and NIVD groups had a significant smaller intervertebral space height as compared to the other two groups, and it substantially decreased over time. Of note, height of intervertebral spaces adjacent to both sides of vertebral body in Mid group seemed to be reduced, whereas height of the intervertebral spaces in the proximity of the drilled hole, but not that distant, shows clear changes in the NIVD group. The quantitative index of intervertebral space height DHI also changed in Mid and NIVD groups, and in particular, it significantly decreased over time, as shown in Figure 2B and Table 2 (*p* < 0.05, differences in DHI between Mid or NIVD group and Normal or Blank group at 1, 2 and 4 weeks). However, no statistically significant difference was observed between Normal and Blank groups (*p* > 0.05). Moreover, after 1 week, the decrease in intervertebral space height in the NIVD group was greater than that measured in the Mid group (mean difference = 2.942, *p* < 0.05, 2 weeks; mean difference = 2.198, *p* < 0.05, 4 weeks).

**TABLE 2:** Simple effect analysis for pairwise comparisons between group and time in disc height index (DHI)

Subjects			Mean difference (I‐J)	*p*	95% confidence interval for difference	
Group	Time (I)	Time (J)			Lower bound	Upper bound
Normal	1w	2w	−0.301	0.884	−1.404	0.802
		4w	−0.379	0.793	−1.482	0.724
	2w	4w	−0.079	0.997	−1.182	1.024
Blank	1w	2w	−0.077	0.998	−1.180	1.026
		4w	−0.407	0.756	−1.510	0.696
	2w	4w	−0.329	0.853	−1.432	0.774
Mid	1w	2w	1.112*	0.047	0.009	2.215
		4w	2.614*	0.000	1.511	3.717
	2w	4w	1.502*	0.004	0.399	2.605
NIVD	1w	2w	3.524*	0.000	2.421	4.627
		4w	4.282*	0.000	3.179	5.385
	2w	4w	0.758	0.270	−0.345	1.861

Time	Group (I)	Group (J)				
1w	Normal	Blank	0.140	1.000	−1.077	1.357
		Mid	1.642*	0.003	0.425	2.860
		NIVD	2.173*	0.000	0.955	3.390
	Blank	Mid	1.502*	0.007	0.285	2.720
		NIVD	2.033*	0.000	0.815	3.250
	Mid	NIVD	0.530	0.819	−0.687	1.748
2w	Normal	Blank	0.363	0.965	−0.854	1.581
		Mid	3.055*	0.000	1.838	4.273
		NIVD	5.997*	0.000	4.780	7.214
	Blank	Mid	2.692*	0.000	1.475	3.909
		NIVD	5.634*	0.000	4.416	6.851
	Mid	NIVD	2.942*	0.000	1.724	4.159
4w	Normal	Blank	0.113	1.000	−1.105	1.330
		Mid	4.636*	0.000	3.419	5.853
		NIVD	6.834*	0.000	5.616	8.051
	Blank	Mid	4.523*	0.000	3.306	5.741
		NIVD	6.721*	0.000	5.504	7.938
	Mid	NIVD	2.198*	0.000	0.980	3.415

Abbreviation: w, week.

*The mean difference is significant at the 0.05 level.

As shown in Figure 2, IVD MRI (panel C) and the total greyscale value of the quantitative index of IVD T2 signal (panel D) decreased dramatically over time in Mid and at higher extent in NIVD groups, as compared to Normal and Blank groups. In particular, at week 1, T2 signal in the NIVD group was significantly lower than that observed in the other groups. Results of simple effect analysis (Table 3) showed that the development of T2 signal was similar to that of DHI of intervertebral space. T2 signal in Mid and NIVD groups decreased markedly over time, and significant differences in greyscale value between Mid or NIVD groups and Normal or Blank groups at 2 and 4 weeks (*p* < 0.05) were observed. Nevertheless, T2 signal in the NIVD group was significantly different from that observed for the other experimental groups at 1 week (mean difference = Normal/Blank/Mid‐NIVD = 7678.549/6545.375/6692.023, *p* < 0.05), and it substantially decreased at 2 and 4 weeks (mean difference = 467.468, *p* = 0.791 for both time‐points).

**TABLE 3:** Simple effect analysis for pairwise comparisons between group and time in MRI

Subjects (grey value)			Mean difference	*P*	95% confidence interval for difference	
Group	Time (I)	Time (J)	(I‐J)		Lower bound	Upper bound
Normal	1w	2w	947.774	0.254	−405.345	2300.893
		4w	456.914	0.802	−896.205	1810.033
	2w	4w	−490.860	0.765	−1843.979	862.259
Blank	1w	2w	170.666	0.986	−1182.453	1523.785
		4w	390.206	0.866	−962.913	1743.325
	2w	4w	219.540	0.972	−1133.579	1572.659
Mid	1w	2w	5634.042*	0.000	4280.923	6987.161
		4w	8884.241*	0.000	7531.122	10237.360
	2w	4w	3250.198*	0.000	1897.079	4603.318
NIVD	1w	2w	2027.015*	0.001	673.896	3380.134
		4w	2494.483*	0.000	1141.364	3847.602
	2w	4w	467.468	0.791	−885.651	1820.587

Time	Group (I)	Group (J)				
1w	Normal	Blank	1133.174	0.242	−360.374	2626.722
		Mid	986.526	0.396	−507.022	2480.073
		NIVD	7678.549*	0.000	6185.001	9172.097
	Blank	Mid	−146.648	1.000	−1640.196	1346.899
		NIVD	6545.375*	0.000	5051.827	8038.823
	Mid	NIVD	6692.023*	0.000	5198.475	8185.571
2w	Normal	Blank	356.066	0.989	−1137.482	1849.613
		Mid	5672.794*	0.000	4179.246	7166.342
		NIVD	8757.790*	0.000	7264.242	10251.338
	Blank	Mid	5316.728*	0.000	3823.181	6810.276
		NIVD	8401.724*	0.000	6908.177	9895.272
	Mid	NIVD	3084.996*	0.000	1591.448	4578.544
4w	Normal	Blank	1066.466	0.306	−427.082	2560.014
		Mid	9413.853*	0.000	7920.305	10907.400
		NIVD	9716.118*	0.000	8222.570	11209.666
	Blank	Mid	8347.387*	0.000	6853.839	9840.934
		NIVD	8649.652*	0.000	7156.104	10143.200
	Mid	NIVD	302.265	0.995	−1191.282	1795.813

Abbreviation: w, week.

*The mean difference is significant at the 0.05 level.

### 3.2 Histological analyses

**FIGURE 3:**
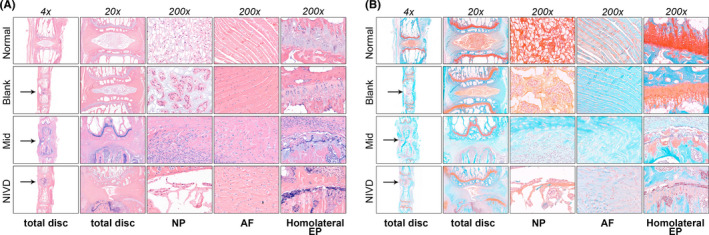
Histological stainings (4 weeks) revealed that the NP disappeared, the AF structure was disordered, the fibres were thickened, large numbers of inflammatory cells infiltrated, and the cartilage EP degenerated and disappeared in Mid and NIVD groups. (A) Haematoxylin‐eosin (HE) staining; (B) safranin O/fast green (SOFG) staining. AF, annulus fibrosus; Homolateral EP, homolateral endplate of the vertebral body; NP, nucleus pulposus. Black arrow indicates drilling location in the vertebral body

Haematoxylin‐eosin staining (Figure 3A) of sections of vertebral body and IVD showed that in rats of Normal and Blank groups, the nucleus pulposus (NP) and the annulus fibre (AF) retained intact morphology. In Mid and NIVD groups, the intervertebral space height decreased, and NP degenerated and replaced by many fibres. AF structure was disordered, with thickened fibres, and highly infiltrated by numerous inflammatory cells. In the Mid group, infiltrating cells were present in both sides, whereas the NIVD group showed inflammatory cells mainly concentrated in the proximity of drilled hole. SOFG staining (Figure 3B) also revealed the above‐mentioned changes, as well as modifications of the cartilage endplate (EP). In particular, in Normal and Blank groups, the cartilage EP was red, with intact structure and tight arrangement of chondrocytes, whereas in Mid and NIVD groups, the cartilage EP on the side of drilled hole was damaged and showed irregular structure and loss of hyaline and chondrocytes, as well as a large number of inflammatory cells.

**FIGURE 4:**
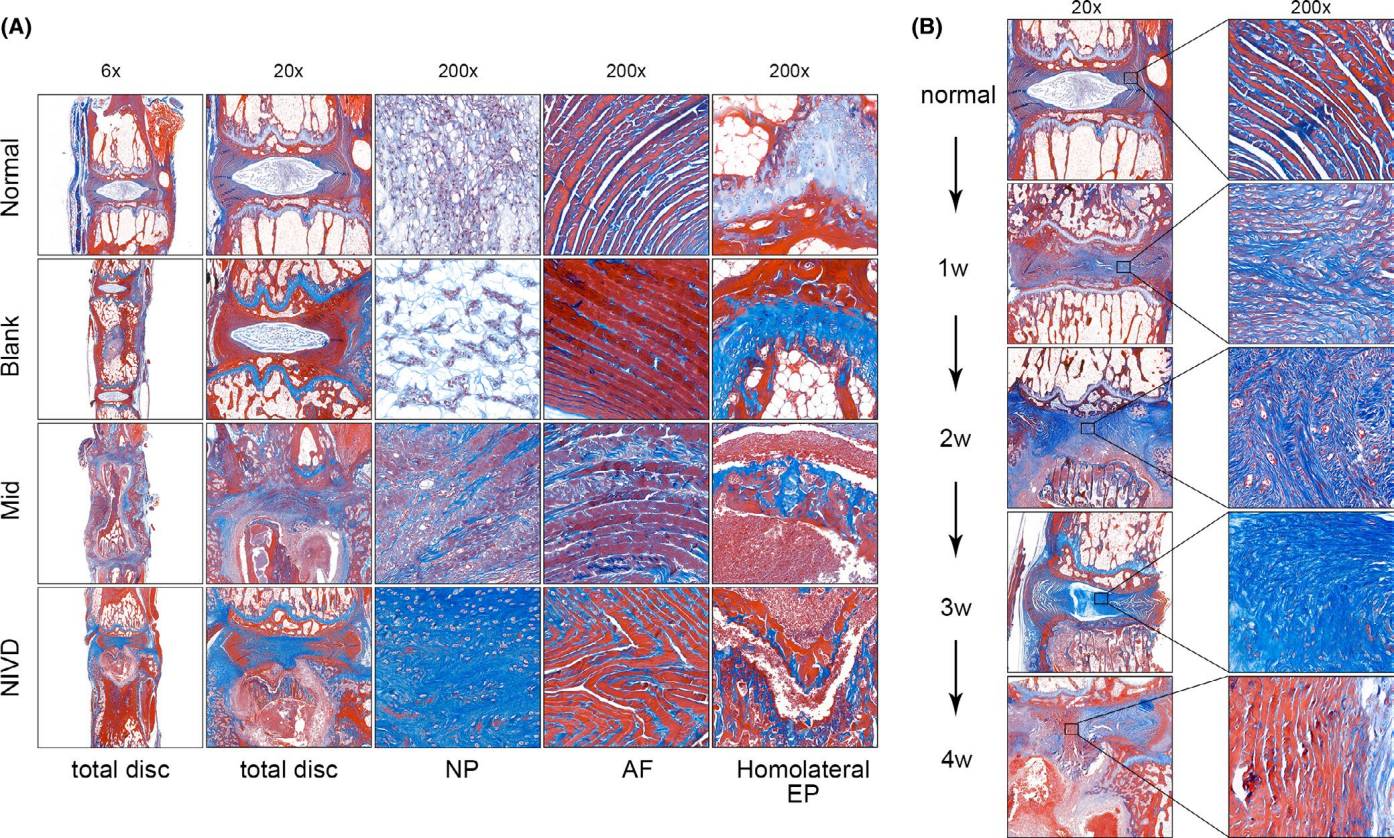
Masson's trichrome staining. (A) In Mid and NIVD groups, NP disappeared, AF structure was disordered, fibres were thickened, a large number of fresh collagen fibres were formed (blue), and cartilage EP degenerated and disappeared (4 weeks); (B) many immature fresh collagen fibres (blue) were formed in the degenerative IVD over time, and gradually matured (red) up to 4 weeks. NP: nucleus pulposus, AF, annulus fibrosus, and Homolateral EP, homolateral endplate of the vertebral body

Masson staining (Figure 4) mainly stained mature collagen fibres in IVD structure,^35^ especially by enhancing contrast. As shown in Figure 4A, in normal IVD, AF and cartilage EP are mostly visible as dark red and light blue, respectively. In Mid and NIVD groups, AF was more disordered, and light blue staining of cartilage EP disappeared. As shown in Figure 4B, blue staining area of the degenerated IVD extended at late time‐point of observation, indicating that immature fresh collagen fibres were gradually formed (immature and mature collagen fibres stain blue and red, respectively). At week 4, the newly formed collagen fibres showed maturation signs.

### 3.3 Immunohistochemistry analysis of IVD‐related molecules (Aggrecan, Col‐II and MMP‐3)

**FIGURE 5:**
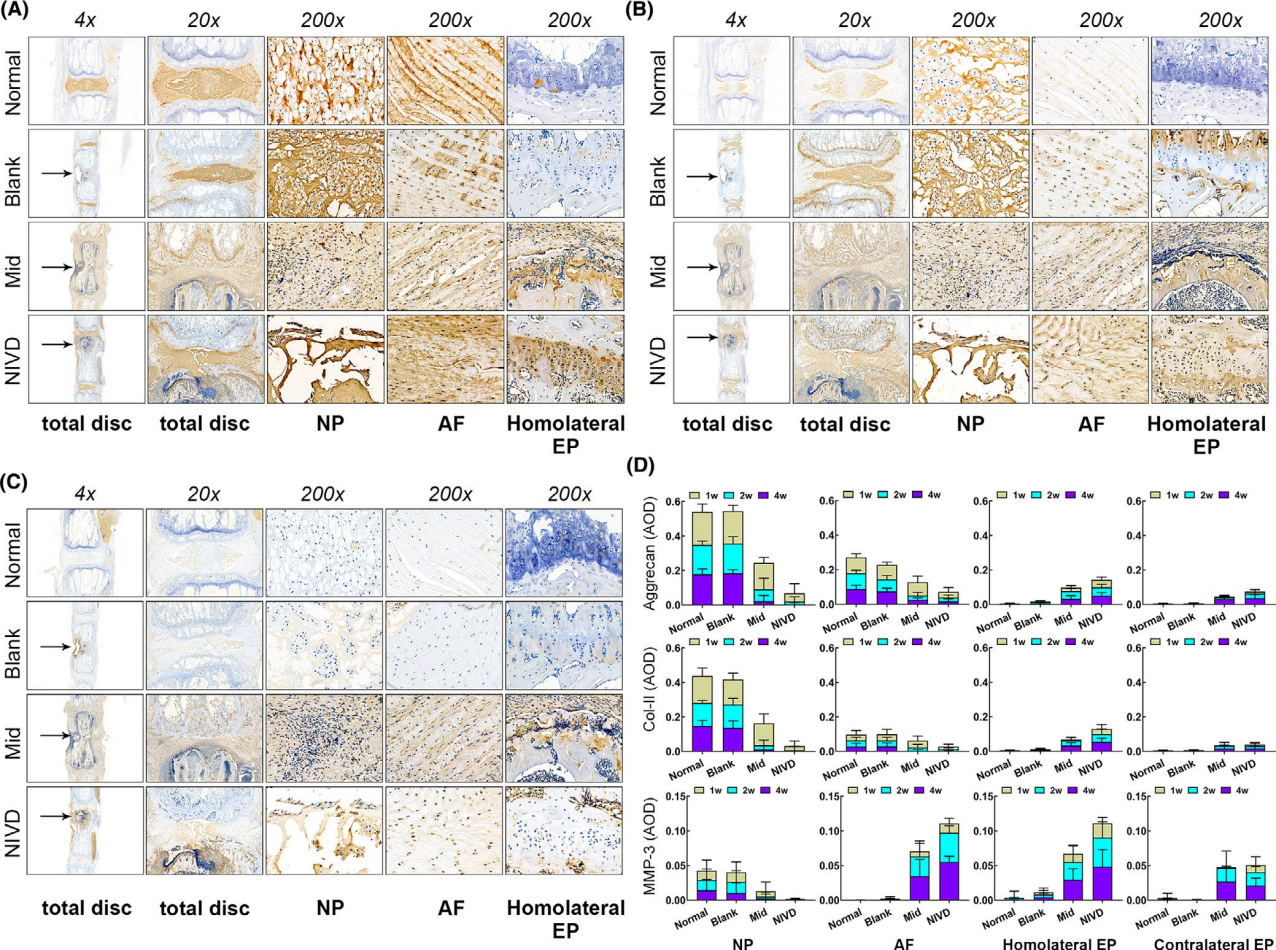
IHC analysis of the IVD‐related markers Aggrecan, Collagen‐II and MMP‐3. (A) Aggrecan; (B) Collagen‐II; (C) MMP‐3; (D) statistical analysis relative to AOD showed that the expressions of Aggrecan and Collagen‐II significantly decreased in IVD or AF, but increased in EP in Mid and NIVD groups. MMP‐3 was highly expressed in Mid and NIVD groups. AF, annulus fibrosus; Homolateral EP: homolateral endplate of vertebral body; NP, nucleus pulposus. Contralateral EP: contralateral endplate of vertebral body. Black arrow indicates drilling location in the vertebral body

Figure 5 shows Aggrecan, Col‐II and MMP‐3 expression in NP, AF and EP in the different experimental groups. In Normal and Blank groups, Aggrecan (panel A) and Col‐II (panel B) were highly expressed in NP and AF and slightly in EP. In parallel, MMP‐3 expression (panel C) was scarce in NP, AF and EP in both groups. In Mid and NIVD groups, Aggrecan and Col‐II decreased in NP and AF and increased in EP, whereas MMP‐3 expression increased in both groups.

Figure 5D shows the statistical analysis relative to AOD measurements, which quantify the expression of each analysed marker index. No significant differences in Aggrecan and Col‐II expressions between Normal and Blank groups at 1, 2 and 4 weeks were observed. In contrast, significant differences were observed in Aggrecan and Col‐II expression when Mid, NIVD and Normal (or Blank) groups were compared (*p* < 0.05). Moreover, Aggrecan and Col‐II expression in NP and AF in Mid and NIVD groups decreased, even though their levels in EP were slightly higher in Mid and NIVD groups than in the Normal group (or Blank group). MMP‐3 expression was higher in Mid and NIVD groups than in the Normal group (or Blank group), in all areas with the exception of NP (it was probably related to the degeneration and disappearance of NP in Mid and NIVD groups). Regardless of marker type and localization, AOD decrease or increase was more evident in the NIVD group than that in the Mid group. The statistical results are shown in Tables [Link], [Link], [Link].

### 3.4 Immunohistochemistry analysis of IL‐1β, IL‐6 and TNF‐α inflammatory molecules

**FIGURE 6:**
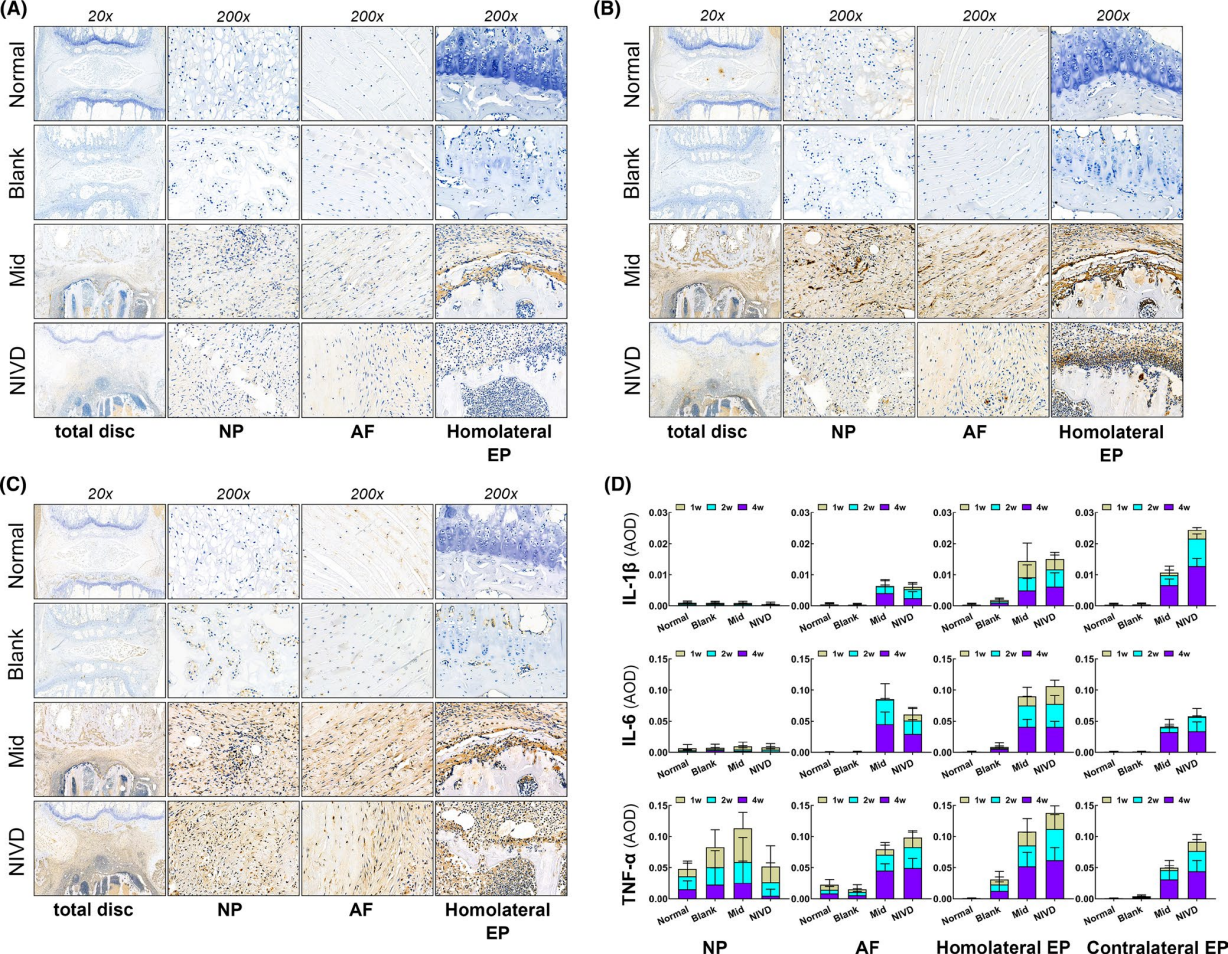
IHC analysis of IL‐1β, IL‐6 and TNF‐α inflammatory cytokines. (A) IL‐1β; (B) IL‐6; (C) TNF‐α; (D) statistical analysis relative to AOD showed that inflammatory marker expression in Mid and NIVD groups significantly increased. AF, annulus fibrosus; Homolateral EP: homolateral endplate of vertebral body; NP, nucleus pulposus. Contralateral EP: contralateral endplate of vertebral body

Figure 6 shows IHC analysis of IL‐1β (panel A), IL‐6 (panel B) and TNF‐α (panel C) in different areas of IVD, as well as statistical analysis data relative to each marker (panel D). The expression of the three inflammatory markers in AF and in the homolateral and contralateral EP of the vertebral body in Mid and NIVD groups was higher than that of the Normal and Blank groups (*p* < 0.05), with IL‐1β expression being lower than IL‐6 and TNF‐α in each group.

However, IL‐1β and IL‐6 expressions were relatively low in NP, with no significant difference among the four groups (*p* > .05). Although the expression of TNF‐α in NP was significantly higher in the Mid group than in Normal and NIVD groups at 1 week (*p* < 0.05), there were no statistically significant differences for the other groups at other time‐points (*p* > 0.05). This was probably due to the degeneration and disappearance of NP tissue in Mid and NIVD groups. The statistical results are shown in Tables [Link], [Link], [Link].

### 3.5 Macrophage infiltration analysis

**FIGURE 7:**
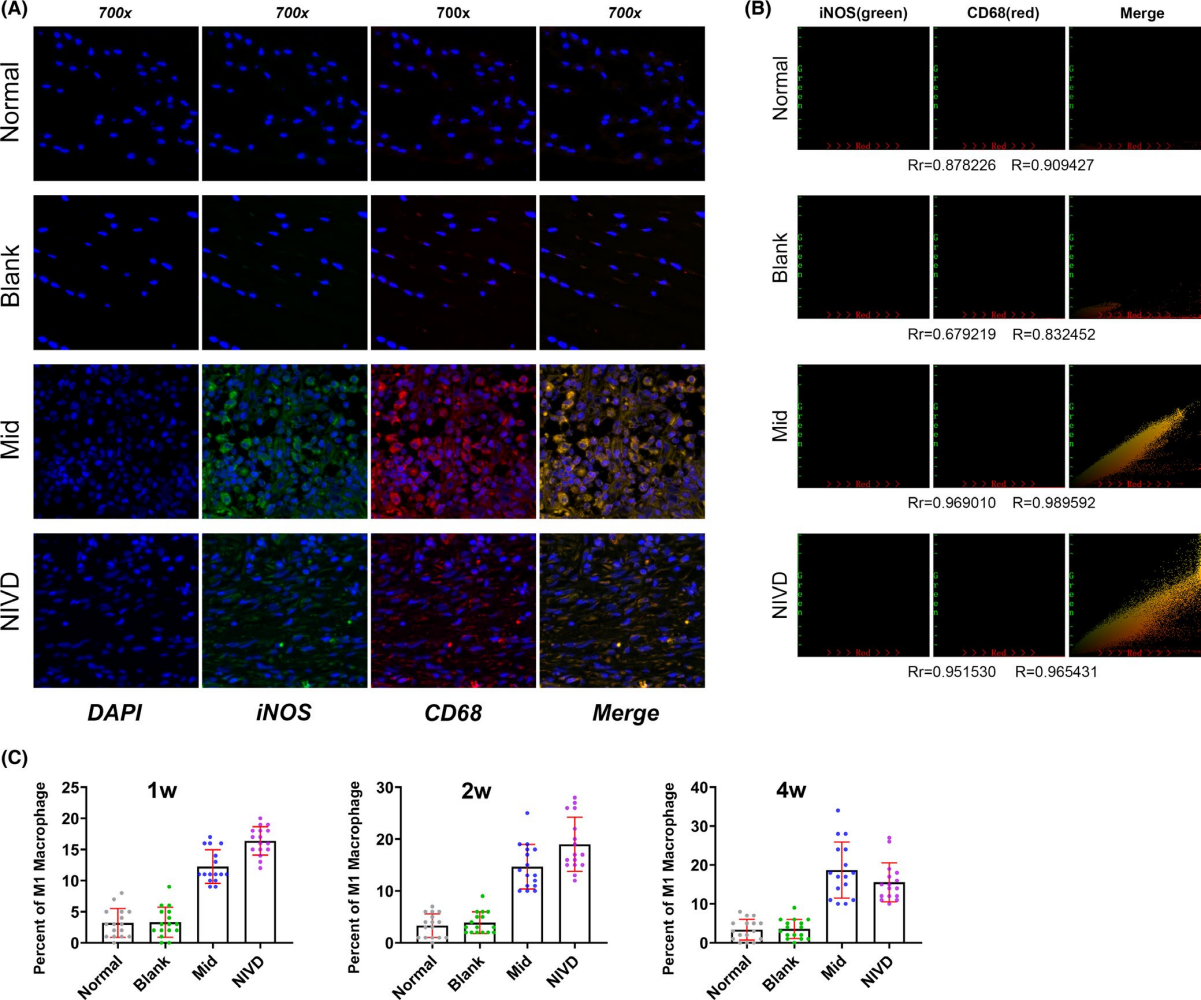
Infiltration of iNOS+and CD68+ M1 macrophages. (A) Double‐IF staining revealed a iNOS and CD68 macrophage infiltrate in Mid and NIVD groups; (B) The co‐location scatter diagram of iNOS (green) and CD68 (red) revealed that iNOS and CD68 co‐localized in Mid and NIVD groups (Rr: Pearson's correlation coefficient; R: overlap coefficient); (C) statistical analysis showed that M1 macrophages were significantly more represented in Mid and NIVD groups than in Normal and Blank groups at the indicated time‐points

We next evaluated M1 macrophage infiltration by analysing iNOS and CD68 specific markers. Double‐IF staining showed a high expression of iNOS and CD68 (Figure 7A) in Mid and NIVD groups. The co‐localization scatter diagram of iNOS and CD68 (Figure 7B) shows that the degree of co‐localization between the two marker indexes was very high in Mid and NIVD groups. The Pearson correlation coefficient (Rr) and overlap coefficient (also called co‐localization coefficient, R) in the Mid group were 0.969010 and 0.989592, respectively. Rr and R in NIVD group were 0.951530 and 0.965431, respectively. Infiltration of M1 macrophages was further quantified, and as shown in Figure 7C, the percentage of M1 macrophages in IVD in Mid and NIVD groups was higher than in Normal and Blank groups at weeks 1, 2 and 4 (1 week: K‐W, *H* = 50.529, *p* < 0.05; 2 weeks: K‐W, *H* = 48.938, *p* < 0.05; 4 weeks: K‐W, *H* = 47.767, *p* < 0.05), and started to slightly decline at week 4.

### 3.6 Apoptosis‐related TUNEL assay

**FIGURE 8:**
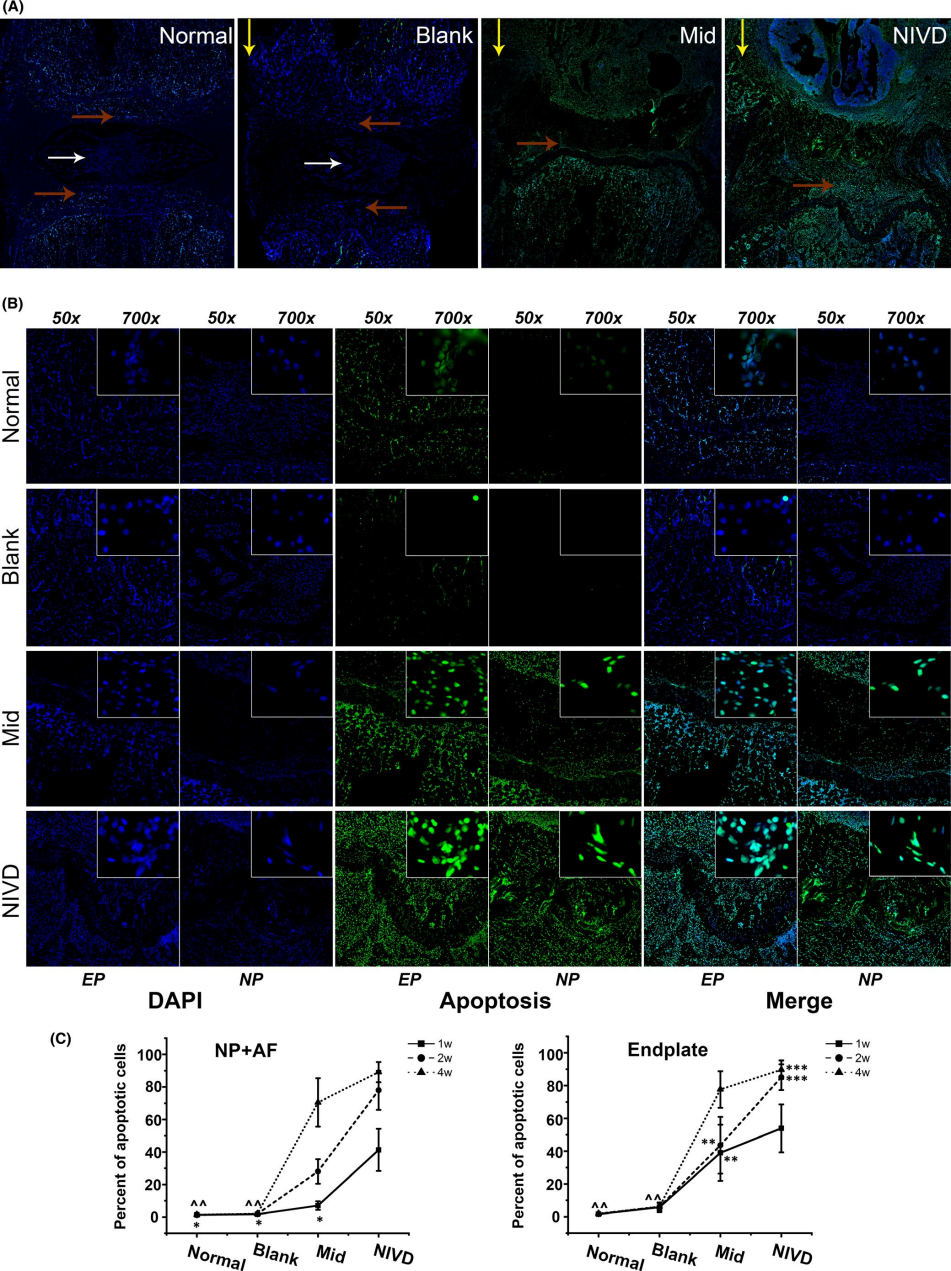
Cell apoptosis was detected by TUNEL assay. (A) The overall pattern of IVD apoptotic cells (green) in each group showed that cell apoptosis was more prominent in Mid and NIVD groups (white arrow: NP, red arrow: cartilage EP, yellow arrow: homolateral to vertebral body); (B) Local apoptosis for each group. The inset panels (Magnified 700x) revealed that cell apoptosis significantly increased in Mid and NIVD groups (NP: nucleus pulposus, AF: annulus fibrosus, EP: endplate); (C) statistical analysis revealed that the number of apoptotic cells was significantly higher in Mid and NIVD groups than that in Normal and Blank groups (^^^^Normal Blank: *p* > 0.05, ^*^Mid‐Normal/Blank: *p* > 0.05; ^**^1–2 weeks (Mid): *p* > 0.05, ^***^2–4 weeks (NIVD): *p* > 0.05. Differences between other unmarked groups at different time‐points were significant, *p* < 0.05)

Figure 8 shows cell apoptosis detected by TUNEL (green) in IVD. As shown in Figure 8A, TUNEL staining revealed a more severe degeneration in Mid and NIVD groups. Hyaline cartilage EP of the vertebral body also degenerated and disappeared (yellow arrow), whereas that present in proximity of the contralateral side degenerated only slightly (red arrow). Figure 8B,C shows the degeneration in NP and AF, as well as in EP. The proportion of degenerative cells was significantly higher in Mid and NIVD groups, and it increased over time, whereas the degeneration was less severe in Normal and Blank groups, with no statistically significant difference between the two groups (*p* > 0.05). At week 1, no significant differences in apoptotic rate between Mid and Normal group (or Blank group) in NP and AF (Mid‐Normal: *p* = 0.165, Mid‐Blank: *p* = 0.224) were observed. On the contrary, in the Mid group, the proportion of apoptotic cells in EP was dramatically higher than that present in the Normal group (or Blank group) at 1 week. In the NIVD group, the apoptosis ratio was higher than that observed in the other groups at all experimental time‐points. Apoptosis rate was particularly high in EP (%: 85.131 ± 7.885) at week 2, and became stable at week 4 (NIVD, 2–4 weeks: *p* = 0.424).

### 3.7 Progression of inflammation and degeneration

**FIGURE 9:**
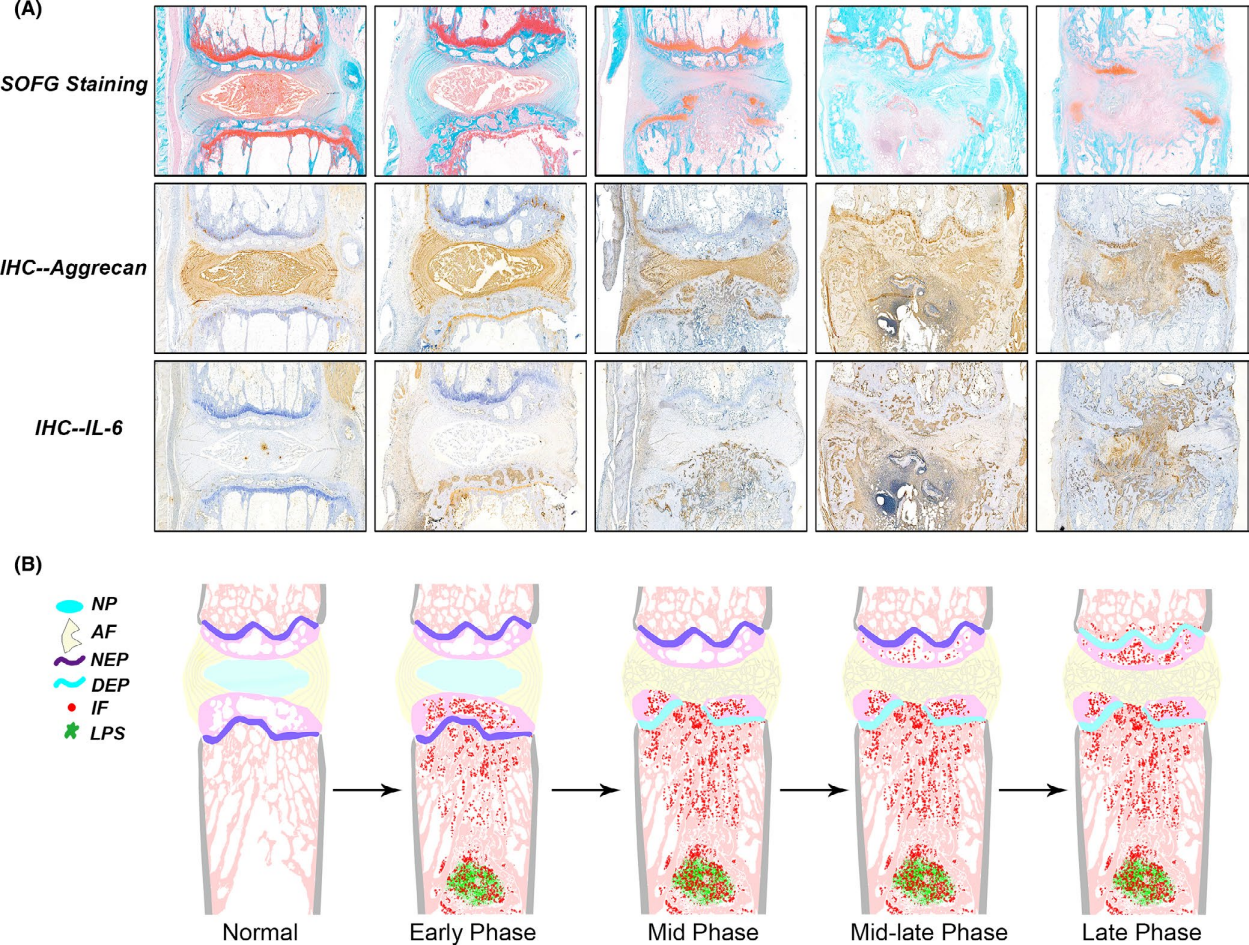
Inflammation signs during development of IVDD in the VI‐IVDD model. (A) Analyses of morphology (SOFG staining), IVD‐related marker (Aggrecan) and inflammatory marker (IL‐6) revealed that inflammation and development of IVDD were time‐dependent. (B) The diagram shows the process of inflammation and degeneration in detail. Early phase: inflammatory factors and cells infiltrate on the side of vertebral body, but no signs of IVDD are present. Mid phase: inflammation reaches IVD, NP degenerates and disappears, AF structure become disordered, and the homolateral EP is damaged. Mid‐late phase: IVDD further worsens and inflammation reaches the contralateral EP. Late phase: severe IVDD and severe damage to the contralateral EP. P‐stage is achieved, and the degeneration shows a stable trend. AF, annulus fibrosus; DEP, degenerative endplate; IF, inflammatory factors/ cells; LPS, lipopolysaccharide; NEP, normal nucleus pulposus; NP, nucleus pulposus

In the early phase of inflammation (within 1 week for the Mid group and about 1–3 days for the NIVD group), we observed that NP, AF and cartilage EP were intact and without signs of degeneration, even though a large number of inflammatory cells infiltrated the LPS‐treated vertebral bodies (Figure 9A). In the middle phase of inflammation (1–1.5 weeks for Mid group and about 1 week for NIVD group), signs of inflammation were evident in NP and AF (Figure 9A). In particular, NP started degeneration process, the AF structure was disordered, and the homolateral cartilage EP of the vertebral body was damaged by inflammation. During the mid‐late phase of inflammation (1.5–3 weeks for the Mid group and 1–2 weeks for the NIVD group), IVDD severity increased, with the inflammation foci spreading into the contralateral EP, even though tissue was not damaged. In the late phase of inflammation (about 3 weeks for the Mid group and about 2 weeks for the NIVD group), IVDD markedly manifested, being intervertebral space height substantially reduced and the contralateral cartilage EP of vertebral body significantly damaged by inflammation.

### 3.8 Activation of the cGAS/STING signalling pathway

**FIGURE 10:**
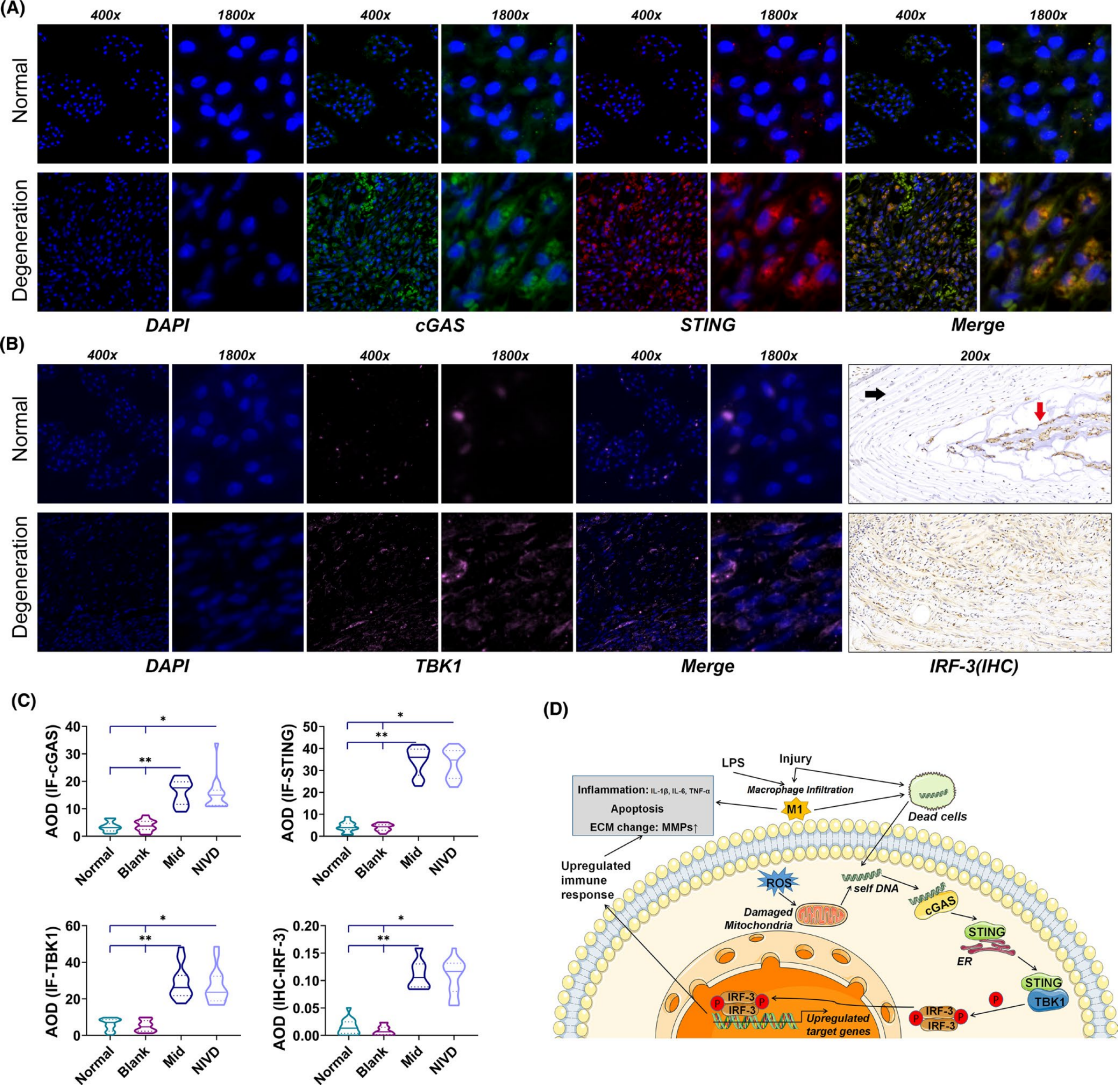
Activation of the cGAS/STING signalling pathway in the VI‐IVDD model. (A) Double‐IF staining showed high cGAS and STING expression, in degenerative IVD. The two molecules co‐localized; (B) IF of TBK1 and IHC of IRF3 showed high expression of TBK1 and IRF3 in AF (black arrow) and in NP (red arrow); (C) statistical analysis of AOD showed that cGAS, STING, TBK1 and IRF3 expressions were higher in Mid and NIVD groups than in Normal and Blank groups (^*^NIVD‐Normal/Blank: *p* < 0.05; ^**^Mid‐Normal/Blank: *p* < 0.05); (D) hypothetical pattern of mechanisms downstream LPS activation of cGAS/STING signalling pathway, triggering inflammation and IVDD development in the VI‐IVDD model (ECM, extracellular matrix; ER, endoplasmic reticulum; ROS, reactive oxygen species and red P: phosphorylation)

Figure 10A shows double‐IF staining of cGAS and STING, two key proteins in the cGAS/STING signalling pathway. We found that cGAS and STING expressions were significantly increased in degenerated IVD. Co‐localization analysis demonstrated that the Pearson correlation coefficient Rr was 0.899142, and the overlap coefficient was 0.952959. TBK1 and IRF3, two molecules involved in cGAS/STING signalling pathway, also substantially increased in IVD (Figure 10B), as assessed by IF and IHC analysis, respectively. Quantitative AOD analysis showed that cGAS, STING, TBK1 and IRF3 expressions in Mid and NIVD groups were significantly higher than those detected in Normal and Blank groups (*H* = 47.517, *p* < 0.05 for cGAS; *H* = 47.350, *p* < 0.05 for STING; *H* = 47.484, *p* < 0.05 for TBK1; *H* = 47.703, *p* < 0.05 for IRF3) (Figure 10C).

## 4 DISCUSSION

Generating an effective and reliable animal model of intervertebral disc degeneration (IVDD) can be helpful for studying pathological mechanisms and signal transduction pathways underlying IVDD condition. IVDD animal models are also important to identify new interventional therapeutic strategies.

In this study, a new type of vertebral inflammation–induced caudal IVDD rat model, the VI‐IVDD model, was established by using LPS. Moreover, two experimental types of VI‐IVDD model were generated, the VI‐IVDD basic and VI‐IVDD acceleration model, corresponding to the Mid group and NIVD group, respectively. The effectiveness and reproducibility of VI‐IVDD model were assessed by studying a number of parameters, including anatomical, histological and cytochemical features, as well as the pathological processes associated with IVDD, such as apoptosis and inflammatory molecular mechanisms.

Currently, the IVD abnormal stress model, such as the IVD compression or animal standing model, and the IVD damage model (ie IVD acupuncture or EP damage model) represent the mostly used IVDD animal models, showing some physiological and pathological elements in common to IVDD.^13^, ^19^, ^20^ These models are easily handled and can also be employed to evaluate surgical procedures.^8^, ^15^, ^22^, ^23^ Although the stress model recapitulates many features of IVDD, it requires long modelling time and high costs, as well as shows low success rate. On the contrary, the damage model can be realized with a high success rate and at low cost, and more importantly is easily operable, so that it is one of the most favourite animal model in many studies on IVDD. However, in this model, the IVD structure is highly damaged, determining a degeneration progress too rapid and irreversible due to strong inflammatory responses in the damaged site.^8^, ^15^, ^23^ The damage model cannot be employed in studies aimed at identifying mechanisms involved in age‐related natural IVDD.

A large body of literature supports that inflammation is a major inducer and accelerator of IVDD pathological processes.^4^, ^36^, ^37^ Inflammation can further affect and damage blood circulation into the disc, thereby impairing oxygen and nutrient supply. As shown in the degeneration progression diagram (Figure 9), inflammation is responsible for irreversible damage of parts of the vertebral body and endplate structure, which is essential for nutrient vehiculation to the disc. As a consequence, the oxygen supply and nutrient availability to the disc are inadequate, and disc degenerates.

Hence, in this study, inflammation was used to induce IVDD, which ensured the success rate of degeneration and shortened the modelling period. Importantly, in order to maintain intact the IVD structure, the trigger point of inflammation was moved to the vertebral body (Figure 1), and LPS used to trigger inflammation. Many studies demonstrated that LPS is a stable, long‐lasting and efficient inflammation trigger,^24^, ^38^, ^39^ determining effects easy to control and possibly not causing systemic inflammatory responses. Moreover, LPS powder is easy to obtain and to implant into the vertebral body of the animals. In our model, LPS was placed in the middle of the vertebral body (Mid group, also called VI‐IVDD basic model) or in proximity of one side of the vertebral body (NIVD group, also called VI‐IVDD acceleration model), thus determining the degeneration of IVDs adjacent to both sides of vertebral body or strictly close to the trigger point, respectively. In the latter type of model, degeneration speed was slightly higher than that obtained in the Mid group, and the adjacent IVD located in the opposite side with respect to the trigger point showed no degeneration. As a result, an innovative and controllable manipulation of degenerative segment (degenerative segment is uncontrollable in standing model) and degeneration speed (degeneration speed is uncontrollable in IVD damage model) of VI‐IVDD was realized.

Radiological images of VI‐IVDD rats show a degenerated IVD segment characterized by a reduced intervertebral space height and low MRI T2 signal.^8^ According to our previous studies, the molybdenum‐target plain X‐ray is more powerful than common plain X‐ray, in terms of image contrast and resolution of microstructures, such as caudal intervertebral space in rats.^22^ Molybdenum‐target plain X‐ray also permitted a better localization of the drilled hole and the trigger point of inflammation in the vertebral body, thus providing a support guaranteeing accuracy of analysis of modelling operation. The VI‐IVDD model (Mid and NIVD groups) showed signs of degeneration of IVD adjacent to the vertebral body (decreased DHI and weakened MRI T2 signal). In contrast, the Blank group (without LPS) did not show the above signs of degeneration, further confirming the importance of inflammation in inducing IVDD. In addition, we found that, in the VI‐IVDD model, the decrease in MRI T2 signal intensity was greater than that of DHI at weeks 2 and 4. In the NIVD group, the decrease in DHI was not evident at week 1, whereas the reduction in MRI T2 signal was remarkable (Figure 2). These data suggest that, in IVDD, the MRI T2 signal changes before intervertebral space modification, which is in line with clinical experience and provides a basis for clinical evaluation.

Histological analysis of VI‐IVDD model (Figures 3 and 4) showed several histomorphological changes of IVDD, including disappearance of NP, degeneration of AF structure, thickening of fibres, damage and loss of cartilage EP, and infiltration of a number of inflammatory cells.^40^ HE staining showed the overall tissue structure and infiltration of inflammatory cells, whereas SOFG was very effective in differential visualization of NP, AF and EP. In particular, EP analysis was important for evaluation of EP degeneration (Figure 3). The IVD collagen maturity was analysed by Masson staining (Figure 4).^35^ We also found that, in the VI‐IVDD model, several immature fresh collagen fibres formed in the IVD, which degenerated over time. Collagen fibre formation gradually occurred from week 1 to 3, to reach a maximum at about week 3. Afterwards, the newly formed collagen fibres matured at 4 weeks.

As a whole, these data show that the process of collagen tissue remodelling is associated with IVDD, a condition that, in VI‐IVDD model, occurs when IVDD reaches a peak plateau stage (P‐stage) at about week 4.

Aggrecan and Col‐II expressions are high in NP and AF of normal IVDs.^8^ IHC (Figure 5) showed that, in the VI‐IVDD model, Aggrecan and Col‐II decreased in NP and AF, but increased in EP. This condition could be related to the remodelling and degeneration of the damaged tissue.^41^ In addition, in our VI‐IVDD model, MMP‐3 expression remarkably increased, together with fresh collagen fibres, as assessed by Masson staining, suggesting that the extracellular matrix was subjected to remodelling.^42^ Hence, it seems that the IVD in the VI‐IVDD model recapitulates the natural degeneration process.

Figures 4 and 5 show the expression of inflammatory factors in the IVD and the infiltration of inflammatory cells in VI‐IVDD model, such as iNOS+and CD68+ M1 macrophages. The presence of the latter is strongly determined by LPS,^32^ which also induces in macrophages the release of several inflammatory cytokines, including IL‐1β, IL‐6 and TNF‐α.^43^ In VI‐IVDD, M1 macrophages infiltrated massively, and inflammatory cytokines were abundantly produced. In the Mid group, inflammatory signs are present in the adjacent IVDs, in both sides of the vertebral body, whereas in the NIVD group, inflammation was mainly concentrated in the adjacent IVD in the proximity of the trigger point of inflammation. Hence, the VI‐IVDD model caused by vertebral inflammation‐induced local inflammation is effective and is realized by controlling the direction of inflammation propagation. Similar to what observed for changes in collagen fibres, we found that although the expressions of inflammatory cytokines in VI‐IVDD model gradually increased from week 1 to 4, and peaked at 4 weeks, the infiltration of M1 macrophages slightly decreased at 4 weeks, and NIVD group showed a more evident decrease than that observed for the Mid group. Therefore, it can be further hypothesized that IVDD reaches the P‐stage end at 4 weeks in VI‐IVDD model and that the degeneration is severe. At this time‐point of observation, although inflammation was persistent, the progression of degeneration decreased thereafter. In addition, the trigger point of inflammation in the NIVD group was closer to IVD, so that it reached P‐stage more quickly in the NIVD group as compared to the Mid group. Thus, in the VI‐IVDD model, degeneration speed can be controlled by triggering inflammation in different positions (Mid group: low speed, NIVD group: high speed).

TUNEL assay (Figure 8) revealed a substantial amount of apoptotic cells in the VI‐IVDD model and that the NIVD group had higher apoptosis rate than the Mid group, and reached P‐stage earlier. Cell apoptosis is an important factor in IVDD,^6^, ^8^, ^37^, ^40^ and our findings further support the effectiveness of the VI‐IVDD model.

In addition, we found that the inflammation spreading is strictly time‐dependent (Figure 9). In fact, during the early phase of inflammation, inflammation signs were limited to homolateral vertebral bodies and reached the contralateral vertebral bodies in the late phase of inflammation. In the late phase of inflammation, IVDD achieved the P‐stage discussed above. In the NIVD group, early‐to‐middle transition phase, as well as P‐stage achievement, was shorter than in the Mid group since the trigger point of inflammation was closer to homolateral IVD. Compared with our model, in the previously mentioned IVD damage model, the degeneration process may initiate starting directly from the middle phase, so that the associated degeneration process is too fast to be studied appropriately (Figure 9B).

The cGAS/STING signalling pathway has been closely related to the activation of inflammatory responses. Cytoplasmic free DNA is recognized as a danger signal by DNA receptor cGAS. The downstream STING acts as an adaptor molecule and then activates the downstream signals TBK1 and IRF3 to produce cytokines stimulating specific immune responses.^44^ Previous studies demonstrated that LPS can induce the production of several inflammatory cytokines by activating the cGAS/STING signalling pathway^25^ and that inflammatory responses activated by the cGAS/STING signalling pathway can in turn induce IVDD.^45^ In this study, we found that cGAS/STING signalling pathway activated by LPS is closely related to IVDD. Figure 10 shows the activation of the cGAS/STING signalling pathway in the VI‐IVDD model and identifies this molecular cascade as the potential mechanism inducing IVDD (Figure 10D). In our model, we hypothesize that LPS is responsible for massive M1 macrophage infiltration and for tissue damage and inflammation. The latter lead, in turn, to cell death and release of free DNA, also from mitochondria, that could trigger downstream signals, such as STING, TBK1 and IRF3, by binding to cGAS and finally up‐regulating immune responses. The up‐regulated immune response further enhance local production of cytokines, which induce cell apoptosis and extracellular matrix remodelling, finally resulting in IVDD.

However, this study shows some limitations: (1) no experimental group treated with anti‐inflammatory molecules delaying the progression of IVDD was set up. In the case of anti‐inflammatory molecules that could delay IVDD in the VI‐IVDD model, it would be important to realize controllable speed of IVDD in VI‐IVDD models; (2) no experimental group treated with inhibitory molecules of cGAS/STING signalling pathway was set up. Future studies exploring a more comprehensive view of mechanisms underlying IVDD, induced by LPS and activating the cGAS/STING signalling pathway, are needed.

Besides, the following minor findings of significance were revealed: (1) during IVDD, the sign of MRI T2 signal change occurred earlier than that of DHI change; (2) the model reached P‐stage at about 4 weeks (maybe earlier in the NIVD group/IVDD acceleration model), after which the severity of degeneration showed a steady trend; (3) a large number of fresh collagen fibres were formed (extracellular matrix remodelling) in the IVD during degeneration, and these newly formed collagen fibres matured gradually when reaching P‐stage.

## 5 CONCLUSIONS

In this study, we describe a new type of vertebral inflammation–induced caudal IVDD rat model (VI‐IVDD), as well as the processes of inflammation and progression of IVDD condition (Figure 9). Our LPS‐inducible model is strictly associated with cGAS/STING signalling pathway activation (Figure 10). Compared with other commonly used IVDD models, this model does not determine direct damage to the IVD structure, which is convenient for studying the mechanism of natural IVDD. Moreover, it has the advantage of being easily handling, having low costs and high success rate, and short modelling period, and achieving controllable degenerative segment and degeneration speed (Mid group: basic IVDD, NIVD group: accelerated IVDD). The effectiveness and repeatability of the VI‐IVDD model were verified by imaging, histomorphology, histochemistry, cytochemistry (inflammation and apoptosis) and molecular mechanism.

Our model could have broad application prospects and could be helpful for unveiling new pathological mechanisms underlying IVDD condition.

## CONFLICT OF INTERESTS

The authors declared no potential conflicts of interest with respect to the research, authorship and/or publication of this article.

## AUTHOR CONTRIBUTIONS

**Qihang Su:** Conceptualization (lead); Formal analysis (lead); Methodology (lead); Project administration (lead); Resources (lead); Visualization (lead); Writing‐original draft (lead). **Qiuchen Cai:** Investigation (equal); Project administration (equal); Resources (equal). **Yongchao Li:** Investigation (equal); Methodology (equal); Resources (equal). **Hengan Ge:** Formal analysis (equal); Investigation (equal); Resources (equal). **Yuanzhen Zhang:** Investigation (equal); Resources (equal). **Yi Zhang:** Investigation (equal); Resources (equal). **Jun Tan:** Investigation (equal); Writing‐review & editing (equal). **Jie Li:** Conceptualization (equal); Investigation (equal); Writing‐review & editing (equal). **Biao Cheng:** Funding acquisition (equal); Supervision (equal); Writing‐review & editing (equal). **Yan Zhang:** Conceptualization (equal); Data curation (equal); Investigation (equal); Writing‐review & editing (equal).

## Supporting information

Figure S1

Table S1

Table S2

Table S3

Table S4

Table S5

Table S6

## Data Availability

All data generated or analysed during this study are included in this published article and available from the corresponding author upon reasonable request.
