# Supplementary material for: A novel rat model of vertebral inflammation–induced intervertebral disc degeneration mediated by activating cGAS/STING molecular pathway
Source: J Cell Mol Med. 2021 Sep 3;25(20):9567–85. doi: 10.1111/jcmm.16898 (PMC8505843; doi:10.1111/jcmm.16898)
Supplement: Supplementary file 2 — Table S1 [file JCMM-25-9567-s004.docx]

| **Table S1. Simple effect analysis for pairwise comparisons between group and time in Aggrecan (IHC)** | | | | | | | |
| --- | --- | --- | --- | --- | --- | --- | --- |
| **Location** | **Subjects(AOD)** | | | **Mean Difference** | ***P*** | **95% Confidence Interval for Difference** | |
|  | ***Group*** | ***Time (I)*** | ***Time (J)*** | (I－J) |  | **Lower Bound** | **Upper Bound** |
| NP | Normal | 1w | 2w | 0.021 | 0.289 | -0.011 | 0.053 |
|  |  |  | 4w | 0.014 | 0.643 | -0.018 | 0.046 |
|  |  | 2w | 4w | -0.007 | 0.925 | -0.039 | 0.025 |
|  | Blank | 1w | 2w | 0.016 | 0.566 | -0.016 | 0.048 |
|  |  |  | 4w | 0.005 | 0.972 | -0.027 | 0.037 |
|  |  | 2w | 4w | -0.010 | 0.822 | -0.042 | 0.022 |
|  | Mid | 1w | 2w | 0.084 | 0.000* | 0.052 | 0.115 |
|  |  |  | 4w | 0.132 | 0.000* | 0.100 | 0.164 |
|  |  | 2w | 4w | 0.048 | 0.001* | 0.016 | 0.080 |
|  | NIVD | 1w | 2w | 0.031 | 0.067 | -0.001 | 0.062 |
|  |  |  | 4w | 0.043 | 0.005* | 0.011 | 0.075 |
|  |  | 2w | 4w | 0.012 | 0.736 | -0.020 | 0.044 |
|  | ***Time*** | ***Group (I)*** | ***Group (J)*** |  |  |  |  |
|  | 1w | Normal | Blank | 0.004 | 1.000 | -0.032 | 0.039 |
|  |  |  | Mid | 0.039 | 0.024* | 0.003 | 0.074 |
|  |  |  | NIVD | 0.145 | 0.000* | 0.110 | 0.180 |
|  |  | Blank | Mid | 0.035 | 0.054 | 0.000 | 0.070 |
|  |  |  | NIVD | 0.141 | 0.000* | 0.106 | 0.176 |
|  |  | Mid | NIVD | 0.106 | 0.000* | 0.071 | 0.142 |
|  | 2w | Normal | Blank | -0.002 | 1.000 | -0.038 | 0.033 |
|  |  |  | Mid | 0.101 | 0.000* | 0.065 | 0.136 |
|  |  |  | NIVD | 0.154 | 0.000* | 0.119 | 0.189 |
|  |  | Blank | Mid | 0.103 | 0.000* | 0.068 | 0.138 |
|  |  |  | NIVD | 0.156 | 0.000* | 0.121 | 0.191 |
|  |  | Mid | NIVD | 0.053 | 0.001* | 0.018 | 0.089 |
|  | 4w | Normal | Blank | -0.005 | 0.999 | -0.040 | 0.030 |
|  |  |  | Mid | 0.157 | 0.000* | 0.121 | 0.192 |
|  |  |  | NIVD | 0.174 | 0.000* | 0.138 | 0.209 |
|  |  | Blank | Mid | 0.162 | 0.000* | 0.126 | 0.197 |
|  |  |  | NIVD | 0.179 | 0.000* | 0.143 | 0.214 |
|  |  | Mid | NIVD | 0.017 | 0.741 | -0.018 | 0.052 |
|  | ***Group*** | ***Time (I)*** | ***Time (J)*** |  |  |  |  |
| AF | Normal | 1w | 2w | 0.002 | 0.996 | -0.016 | 0.019 |
|  |  |  | 4w | 0.004 | 0.939 | -0.014 | 0.022 |
|  |  | 2w | 4w | 0.002 | 0.986 | -0.016 | 0.020 |
|  | Blank | 1w | 2w | 0.014 | 0.163 | -0.004 | 0.032 |
|  |  |  | 4w | 0.009 | 0.552 | -0.009 | 0.027 |

|  |  | 2w | 4w | -0.005 | 0.854 | -0.023 | 0.013 |
| --- | --- | --- | --- | --- | --- | --- | --- |
|  | Mid | 1w | 2w | 0.051 | 0.000* | 0.033 | 0.069 |
|  |  |  | 4w | 0.052 | 0.000* | 0.034 | 0.070 |
|  |  | 2w | 4w | 0.001 | 1.000 | -0.017 | 0.018 |
|  | NIVD | 1w | 2w | 0.010 | 0.417 | -0.008 | 0.028 |
|  |  |  | 4w | 0.015 | 0.110 | -0.002 | 0.033 |
|  |  | 2w | 4w | 0.005 | 0.866 | -0.013 | 0.023 |
|  | ***Time*** | ***Group (I)*** | ***Group (J)*** |  |  |  |  |
|  | 1w | Normal | Blank | 0.008 | 0.829 | -0.011 | 0.028 |
|  |  |  | Mid | 0.015 | 0.213 | -0.004 | 0.035 |
|  |  |  | NIVD | 0.060 | 0.000* | 0.040 | 0.079 |
|  |  | Blank | Mid | 0.007 | 0.925 | -0.013 | 0.027 |
|  |  |  | NIVD | 0.051 | 0.000* | 0.031 | 0.071 |
|  |  | Mid | NIVD | 0.044 | 0.000* | 0.024 | 0.064 |
|  | 2w | Normal | Blank | 0.021 | 0.029* | 0.001 | 0.041 |
|  |  |  | Mid | 0.065 | 0.000* | 0.045 | 0.085 |
|  |  |  | NIVD | 0.068 | 0.000* | 0.049 | 0.088 |
|  |  | Blank | Mid | 0.044 | 0.000* | 0.024 | 0.064 |
|  |  |  | NIVD | 0.047 | 0.000* | 0.028 | 0.067 |
|  |  | Mid | NIVD | 0.003 | 0.999 | -0.016 | 0.023 |
|  | 4w | Normal | Blank | 0.013 | 0.356 | -0.006 | 0.033 |
|  |  |  | Mid | 0.063 | 0.000* | 0.044 | 0.083 |
|  |  |  | NIVD | 0.071 | 0.000* | 0.051 | 0.091 |
|  |  | Blank | Mid | 0.050 | 0.000* | 0.030 | 0.070 |
|  |  |  | NIVD | 0.058 | 0.000* | 0.038 | 0.077 |
|  |  | Mid | NIVD | 0.008 | 0.874 | -0.012 | 0.028 |
|  | ***Group*** | ***Time (I)*** | ***Time (J)*** |  |  |  |  |
| Homo-EP | Normal | 1w | 2w | 0.001 | 0.997 | -0.009 | 0.011 |
|  |  |  | 4w | -0.009 | 1.000 | -0.010 | 0.010 |
|  |  | 2w | 4w | -0.001 | 0.997 | -0.011 | 0.009 |
|  | Blank | 1w | 2w | -0.003 | 0.833 | -0.013 | 0.007 |
|  |  |  | 4w | -0.010 | 1.000 | -0.010 | 0.010 |
|  |  | 2w | 4w | 0.003 | 0.834 | -0.007 | 0.013 |
|  | Mid | 1w | 2w | -0.026 | 0.000* | -0.036 | -0.016 |
|  |  |  | 4w | -0.011 | 0.029* | -0.021 | -0.001 |
|  |  | 2w | 4w | 0.015 | 0.002* | 0.004 | 0.025 |
|  | NIVD | 1w | 2w | -0.005 | 0.578 | -0.015 | 0.005 |
|  |  |  | 4w | -0.006 | 0.335 | -0.017 | 0.004 |
|  |  | 2w | 4w | -0.002 | 0.974 | -0.012 | 0.009 |
|  | ***Time*** | ***Group (I)*** | ***Group (J)*** |  |  |  |  |
|  | 1w | Normal | Blank | -0.003 | 0.990 | -0.014 | 0.009 |
|  |  |  | Mid | -0.018 | 0.000* | -0.029 | -0.007 |

|  |  |  | NIVD | -0.042 | 0.000* | -0.053 | -0.030 |
| --- | --- | --- | --- | --- | --- | --- | --- |
|  |  | Blank | Mid | -0.016 | 0.002* | -0.027 | -0.004 |
|  |  |  | NIVD | -0.039 | 0.000* | -0.050 | -0.028 |
|  |  | Mid | NIVD | -0.023 | 0.000* | -0.035 | -0.012 |
|  | 2w | Normal | Blank | -0.007 | 0.536 | -0.018 | 0.005 |
|  |  |  | Mid | -0.045 | 0.000* | -0.056 | -0.033 |
|  |  |  | NIVD | -0.047 | 0.000* | -0.059 | -0.036 |
|  |  | Blank | Mid | -0.038 | 0.000* | -0.049 | -0.027 |
|  |  |  | NIVD | -0.041 | 0.000* | -0.052 | -0.030 |
|  |  | Mid | NIVD | -0.003 | 0.989 | -0.014 | 0.009 |
|  | 4w | Normal | Blank | -0.003 | 0.990 | -0.014 | 0.009 |
|  |  |  | Mid | -0.029 | 0.000* | -0.040 | -0.018 |
|  |  |  | NIVD | -0.048 | 0.000* | -0.059 | -0.037 |
|  |  | Blank | Mid | -0.027 | 0.000* | -0.038 | -0.015 |
|  |  |  | NIVD | -0.046 | 0.000* | -0.057 | -0.034 |
|  |  | Mid | NIVD | -0.019 | 0.000* | -0.030 | -0.008 |
|  | ***Group*** | ***Time (I)*** | ***Time (J)*** |  |  |  |  |
| Contra-EP | Normal | 1w | 2w | -0.015 | 1.000 | -0.010 | 0.010 |
|  |  |  | 4w | 0.000 | 1.000 | -0.010 | 0.011 |
|  |  | 2w | 4w | 0.000 | 1.000 | -0.010 | 0.011 |
|  | Blank | 1w | 2w | 0.002 | 0.962 | -0.009 | 0.012 |
|  |  |  | 4w | 0.001 | 0.998 | -0.010 | 0.011 |
|  |  | 2w | 4w | -0.001 | 0.990 | -0.012 | 0.009 |
|  | Mid | 1w | 2w | -0.005 | 0.619 | -0.015 | 0.006 |
|  |  |  | 4w | -0.026 | 0.000* | -0.036 | -0.016 |
|  |  | 2w | 4w | -0.021 | 0.000* | -0.032 | -0.011 |
|  | NIVD | 1w | 2w | -0.017 | 0.000* | -0.028 | -0.007 |
|  |  |  | 4w | -0.025 | 0.000* | -0.036 | -0.015 |
|  |  | 2w | 4w | -0.008 | 0.214 | -0.018 | 0.003 |
|  | ***Time*** | ***Group (I)*** | ***Group (J)*** |  |  |  |  |
|  | 1w | Normal | Blank | -0.001 | 1.000 | -0.012 | 0.011 |
|  |  |  | Mid | -0.003 | 0.986 | -0.014 | 0.009 |
|  |  |  | NIVD | -0.009 | 0.234 | -0.020 | 0.003 |
|  |  | Blank | Mid | -0.002 | 0.998 | -0.013 | 0.010 |
|  |  |  | NIVD | -0.008 | 0.359 | -0.019 | 0.004 |
|  |  | Mid | NIVD | -0.006 | 0.678 | -0.017 | 0.006 |
|  | 2w | Normal | Blank | 0.001 | 1.000 | -0.011 | 0.012 |
|  |  |  | Mid | -0.008 | 0.398 | -0.019 | 0.004 |
|  |  |  | NIVD | -0.026 | 0.000* | -0.038 | -0.015 |
|  |  | Blank | Mid | -0.009 | 0.264 | -0.020 | 0.003 |
|  |  |  | NIVD | -0.027 | 0.000* | -0.039 | -0.016 |
|  |  | Mid | NIVD | -0.019 | 0.000* | -0.030 | -0.007 |
|  | 4w | Normal | Blank | 0.000 | 1.000 | -0.012 | 0.011 |
|  |  |  | Mid | -0.029 | 0.000* | -0.041 | -0.018 |
|  |  |  | NIVD | -0.034 | 0.000* | -0.046 | -0.023 |
|  |  | Blank | Mid | -0.029 | 0.000* | -0.040 | -0.017 |
|  |  |  | NIVD | -0.034 | 0.000* | -0.045 | -0.022 |
|  |  | Mid | NIVD | -0.005 | 0.815 | -0.017 | 0.006 |
| *The mean difference is significant at the 0.05 level. | | | | | | | |
