# Supplementary material for: A novel rat model of vertebral inflammation–induced intervertebral disc degeneration mediated by activating cGAS/STING molecular pathway
Source: J Cell Mol Med. 2021 Sep 3;25(20):9567–85. doi: 10.1111/jcmm.16898 (PMC8505843; doi:10.1111/jcmm.16898)
Supplement: Supplementary file 3 — Table S2 [file JCMM-25-9567-s001.docx]

| **Table S2. Simple effect analysis for pairwise comparisons between group and time in Col-II (IHC)** | | | | | | | |
| --- | --- | --- | --- | --- | --- | --- | --- |
| **Location** | **Subjects(AOD)** | | | **Mean Difference** | ***P*** | **95% Confidence Interval for Difference** | |
|  | ***Group*** | ***Time (I)*** | ***Time (J)*** | **(I**－**J)** |  | **Lower Bound** | **Upper Bound** |
| NP | Normal | 1w | 2w | 0.024 | 0.133 | -0.005 | 0.053 |
|  |  |  | 4w | 0.010 | 0.771 | -0.018 | 0.039 |
|  |  | 2w | 4w | -0.014 | 0.589 | -0.042 | 0.015 |
|  | Blank | 1w | 2w | 0.010 | 0.797 | -0.019 | 0.039 |
|  |  |  | 4w | 0.009 | 0.851 | -0.020 | 0.037 |
|  |  | 2w | 4w | -0.001 | 1.000 | -0.030 | 0.028 |
|  | Mid | 1w | 2w | 0.104 | 0.000* | 0.076 | 0.133 |
|  |  |  | 4w | 0.117 | 0.000* | 0.088 | 0.145 |
|  |  | 2w | 4w | 0.012 | 0.660 | -0.016 | 0.041 |
|  | NIVD | 1w | 2w | 0.018 | 0.342 | -0.011 | 0.047 |
|  |  |  | 4w | 0.024 | 0.135 | -0.005 | 0.053 |
|  |  | 2w | 4w | 0.006 | 0.950 | -0.023 | 0.034 |
|  | ***Time*** | ***Group (I)*** | ***Group (J)*** |  |  |  |  |
|  | 1w | Normal | Blank | 0.012 | 0.885 | -0.019 | 0.044 |
|  |  |  | Mid | 0.030 | 0.082 | -0.002 | 0.061 |
|  |  |  | NIVD | 0.133 | 0.000* | 0.101 | 0.165 |
|  |  | Blank | Mid | 0.017 | 0.625 | -0.015 | 0.049 |
|  |  |  | NIVD | 0.121 | 0.000* | 0.089 | 0.152 |
|  |  | Mid | NIVD | 0.103 | 0.000* | 0.072 | 0.135 |
|  | 2w | Normal | Blank | -0.002 | 1.000 | -0.033 | 0.030 |
|  |  |  | Mid | 0.110 | 0.000* | 0.078 | 0.142 |
|  |  |  | NIVD | 0.127 | 0.000* | 0.095 | 0.159 |
|  |  | Blank | Mid | 0.112 | 0.000* | 0.080 | 0.143 |
|  |  |  | NIVD | 0.129 | 0.000* | 0.097 | 0.161 |
|  |  | Mid | NIVD | 0.017 | 0.624 | -0.014 | 0.049 |
|  | 4w | Normal | Blank | 0.011 | 0.940 | -0.021 | 0.042 |
|  |  |  | Mid | 0.136 | 0.000* | 0.104 | 0.167 |
|  |  |  | NIVD | 0.146 | 0.000* | 0.115 | 0.178 |
|  |  | Blank | Mid | 0.125 | 0.000* | 0.093 | 0.157 |
|  |  |  | NIVD | 0.136 | 0.000* | 0.104 | 0.167 |
|  |  | Mid | NIVD | 0.011 | 0.941 | -0.021 | 0.042 |
|  | ***Group*** | ***Time (I)*** | ***Time (J)*** |  |  |  |  |
| AF | Normal | 1w | 2w | -0.001 | 0.998 | -0.017 | 0.015 |
|  |  |  | 4w | 0.006 | 0.697 | -0.009 | 0.022 |
|  |  | 2w | 4w | 0.008 | 0.583 | -0.008 | 0.023 |
|  | Blank | 1w | 2w | 0.003 | 0.967 | -0.013 | 0.019 |
|  |  |  | 4w | 0.007 | 0.621 | -0.009 | 0.023 |
|  |  | 2w | 4w | 0.004 | 0.875 | -0.011 | 0.020 |
|  | Mid | 1w | 2w | 0.020 | 0.007* | 0.004 | 0.036 |
|  |  |  | 4w | 0.036 | 0.000* | 0.020 | 0.051 |
|  |  | 2w | 4w | 0.015 | 0.061 | 0.000 | 0.031 |
|  | NIVD | 1w | 2w | 0.007 | 0.689 | -0.009 | 0.022 |
|  |  |  | 4w | 0.012 | 0.187 | -0.004 | 0.028 |
|  |  | 2w | 4w | 0.006 | 0.777 | -0.010 | 0.021 |
|  | ***Time*** | ***Group (I)*** | ***Group (J)*** |  |  |  |  |
|  | 1w | Normal | Blank | -0.002 | 1.000 | -0.020 | 0.015 |
|  |  |  | Mid | -0.005 | 0.972 | -0.022 | 0.012 |
|  |  |  | NIVD | 0.019 | 0.028* | 0.001 | 0.036 |
|  |  | Blank | Mid | -0.003 | 0.999 | -0.020 | 0.015 |
|  |  |  | NIVD | 0.021 | 0.010* | 0.004 | 0.039 |
|  |  | Mid | NIVD | 0.024 | 0.002* | 0.006 | 0.041 |
|  | 2w | Normal | Blank | 0.002 | 1.000 | -0.016 | 0.019 |
|  |  |  | Mid | 0.016 | 0.082 | -0.001 | 0.034 |
|  |  |  | NIVD | 0.026 | 0.001* | 0.009 | 0.044 |
|  |  | Blank | Mid | 0.015 | 0.148 | -0.003 | 0.032 |
|  |  |  | NIVD | 0.025 | 0.001* | 0.007 | 0.042 |
|  |  | Mid | NIVD | 0.010 | 0.550 | -0.007 | 0.028 |
|  | 4w | Normal | Blank | -0.002 | 1.000 | -0.019 | 0.016 |
|  |  |  | Mid | 0.024 | 0.002* | 0.007 | 0.042 |
|  |  |  | NIVD | 0.025 | 0.002* | 0.007 | 0.042 |
|  |  | Blank | Mid | 0.026 | 0.001* | 0.008 | 0.043 |
|  |  |  | NIVD | 0.026 | 0.001* | 0.009 | 0.044 |
|  |  | Mid | NIVD | 0.000 | 1.000 | -0.017 | 0.018 |
|  | ***Group*** | ***Time (I)*** | ***Time (J)*** |  |  |  |  |
| Homo-EP | Normal | 1w | 2w | 0.000 | 1.000 | -0.014 | 0.013 |
|  |  |  | 4w | 0.000 | 1.000 | -0.014 | 0.013 |
|  |  | 2w | 4w | 0.000 | 1.000 | -0.013 | 0.013 |
|  | Blank | 1w | 2w | -0.039 | 1.000 | -0.013 | 0.013 |
|  |  |  | 4w | -0.003 | 0.953 | -0.016 | 0.011 |
|  |  | 2w | 4w | -0.003 | 0.956 | -0.016 | 0.011 |
|  | Mid | 1w | 2w | -0.023 | 0.000* | -0.036 | -0.010 |
|  |  |  | 4w | -0.026 | 0.000* | -0.040 | -0.013 |
|  |  | 2w | 4w | -0.003 | 0.918 | -0.016 | 0.010 |
|  | NIVD | 1w | 2w | -0.018 | 0.004* | -0.031 | -0.005 |
|  |  |  | 4w | -0.025 | 0.000* | -0.038 | -0.012 |
|  |  | 2w | 4w | -0.007 | 0.479 | -0.021 | 0.006 |
|  | ***Time*** | ***Group (I)*** | ***Group (J)*** |  |  |  |  |
|  | 1w | Normal | Blank | -0.002 | 1.000 | -0.016 | 0.013 |
|  |  |  | Mid | -0.005 | 0.954 | -0.019 | 0.010 |
|  |  |  | NIVD | -0.027 | 0.000* | -0.042 | -0.012 |
|  |  | Blank | Mid | -0.003 | 0.994 | -0.018 | 0.012 |
|  |  |  | NIVD | -0.025 | 0.000* | -0.040 | -0.011 |
|  |  | Mid | NIVD | -0.022 | 0.000* | -0.037 | -0.008 |
|  | 2w | Normal | Blank | -0.001 | 1.000 | -0.016 | 0.013 |
|  |  |  | Mid | -0.028 | 0.000* | -0.042 | -0.013 |
|  |  |  | NIVD | -0.045 | 0.000* | -0.059 | -0.030 |
|  |  | Blank | Mid | -0.026 | 0.000* | -0.041 | -0.012 |
|  |  |  | NIVD | -0.043 | 0.000* | -0.058 | -0.029 |
|  |  | Mid | NIVD | -0.017 | 0.014* | -0.032 | -0.002 |
|  | 4w | Normal | Blank | -0.004 | 0.985 | -0.018 | 0.011 |
|  |  |  | Mid | -0.031 | 0.000* | -0.045 | -0.016 |
|  |  |  | NIVD | -0.052 | 0.000* | -0.066 | -0.037 |
|  |  | Blank | Mid | -0.027 | 0.000* | -0.042 | -0.012 |
|  |  |  | NIVD | -0.048 | 0.000* | -0.063 | -0.033 |
|  |  | Mid | NIVD | -0.021 | 0.001* | -0.036 | -0.006 |
|  | ***Group*** | ***Time (I)*** | ***Time (J)*** |  |  |  |  |
| Contra-EP | Normal | 1w | 2w | 0.000 | 1.000 | -0.009 | 0.008 |
|  |  |  | 4w | 0.000 | 0.999 | -0.009 | 0.008 |
|  |  | 2w | 4w | 0.000 | 1.000 | -0.009 | 0.008 |
|  | Blank | 1w | 2w | 0.001 | 0.998 | -0.008 | 0.009 |
|  |  |  | 4w | 0.002 | 0.949 | -0.007 | 0.010 |
|  |  | 2w | 4w | 0.001 | 0.984 | -0.007 | 0.010 |
|  | Mid | 1w | 2w | -0.015 | 0.000* | -0.024 | -0.007 |
|  |  |  | 4w | -0.015 | 0.000* | -0.023 | -0.006 |
|  |  | 2w | 4w | 0.000 | 0.999 | -0.008 | 0.009 |
|  | NIVD | 1w | 2w | -0.008 | 0.053 | -0.017 | 0.054 |
|  |  |  | 4w | -0.009 | 0.029* | -0.018 | -0.001 |
|  |  | 2w | 4w | -0.001 | 0.995 | -0.009 | 0.008 |
|  | ***Time*** | ***Group (I)*** | ***Group (J)*** |  |  |  |  |
|  | 1w | Normal | Blank | -0.001 | 0.999 | -0.011 | 0.008 |
|  |  |  | Mid | 0.000 | 1.000 | -0.009 | 0.010 |
|  |  |  | NIVD | -0.006 | 0.510 | -0.015 | 0.004 |
|  |  | Blank | Mid | 0.002 | 0.997 | -0.008 | 0.011 |
|  |  |  | NIVD | -0.004 | 0.787 | -0.014 | 0.005 |
|  |  | Mid | NIVD | -0.006 | 0.435 | -0.015 | 0.003 |
|  | 2w | Normal | Blank | -0.001 | 1.000 | -0.010 | 0.009 |
|  |  |  | Mid | -0.015 | 0.000* | -0.024 | -0.005 |
|  |  |  | NIVD | -0.014 | 0.001* | -0.023 | -0.004 |
|  |  | Blank | Mid | -0.014 | 0.000* | -0.024 | -0.005 |
|  |  |  | NIVD | -0.013 | 0.001* | -0.023 | -0.004 |
|  |  | Mid | NIVD | 0.001 | 1.000 | -0.008 | 0.010 |
|  | 4w | Normal | Blank | 0.001 | 1.000 | -0.009 | 0.010 |
|  |  |  | Mid | -0.014 | 0.001* | -0.023 | -0.005 |
|  |  |  | NIVD | -0.014 | 0.000* | -0.024 | -0.005 |
|  |  | Blank | Mid | -0.015 | 0.000* | -0.024 | -0.006 |
|  |  |  | NIVD | -0.015 | 0.000* | -0.024 | -0.006 |
|  |  | Mid | NIVD | 0.000 | 1.000 | -0.010 | 0.009 |
| *The mean difference is significant at the 0.05 level. | | | | | | | |
