# Supplementary material for: A novel rat model of vertebral inflammation–induced intervertebral disc degeneration mediated by activating cGAS/STING molecular pathway
Source: J Cell Mol Med. 2021 Sep 3;25(20):9567–85. doi: 10.1111/jcmm.16898 (PMC8505843; doi:10.1111/jcmm.16898)
Supplement: Supplementary file 5 — Table S4 [file JCMM-25-9567-s006.docx]

| **Table S4. Simple effect analysis for pairwise comparisons between group and time in IL-1β (IHC)** | | | | | | | |
| --- | --- | --- | --- | --- | --- | --- | --- |
| **Location** | **Subjects(AOD)** | | | **Mean Difference** | ***P*** | **95% Confidence Interval for Difference** | |
|  | ***Group*** | ***Time (I)*** | ***Time (J)*** | (I**－**J) |  | **Lower Bound** | **Upper Bound** |
| NP | Normal | 1w | 2w | -0.044 | 0.989 | -0.001 | 0.000 |
|  |  |  | 4w | -0.019 | 0.999 | -0.001 | 0.001 |
|  |  | 2w | 4w | 0.025 | 0.998 | -0.001 | 0.001 |
|  | Blank | 1w | 2w | -0.038 | 0.993 | -0.001 | 0.001 |
|  |  |  | 4w | -0.027 | 0.997 | -0.001 | 0.001 |
|  |  | 2w | 4w | 0.011 | 1.000 | -0.001 | 0.001 |
|  | Mid | 1w | 2w | -0.021 | 0.999 | -0.001 | 0.001 |
|  |  |  | 4w | 0.022 | 0.999 | -0.001 | 0.001 |
|  |  | 2w | 4w | 0.043 | 0.990 | 0.000 | 0.001 |
|  | NIVD | 1w | 2w | 0.000 | 0.686 | 0.000 | 0.001 |
|  |  |  | 4w | 0.000 | 0.848 | 0.000 | 0.001 |
|  |  | 2w | 4w | -0.042 | 0.991 | -0.001 | 0.000 |
|  | ***Time*** | ***Group (I)*** | ***Group (J)*** |  |  |  |  |
|  | 1w | Normal | Blank | 0.013 | 1.000 | -0.001 | 0.001 |
|  |  |  | Mid | 0.014 | 1.000 | -0.001 | 0.001 |
|  |  |  | NIVD | 0.017 | 1.000 | -0.001 | 0.001 |
|  |  | Blank | Mid | -0.009 | 1.000 | -0.001 | 0.001 |
|  |  |  | NIVD | -0.017 | 1.000 | -0.001 | 0.001 |
|  |  | Mid | NIVD | -0.008 | 1.000 | -0.001 | 0.001 |
|  | 2w | Normal | Blank | 0.019 | 1.000 | -0.001 | 0.001 |
|  |  |  | Mid | 0.027 | 1.000 | -0.001 | 0.001 |
|  |  |  | NIVD | 0.000 | 0.761 | 0.000 | 0.001 |
|  |  | Blank | Mid | 0.008 | 1.000 | -0.001 | 0.001 |
|  |  |  | NIVD | 0.000 | 0.835 | 0.000 | 0.001 |
|  |  | Mid | NIVD | 0.000 | 0.863 | 0.000 | 0.001 |
|  | 4w | Normal | Blank | 0.016 | 1.000 | -0.001 | 0.001 |
|  |  |  | Mid | 0.044 | 1.000 | -0.001 | 0.001 |
|  |  |  | NIVD | 0.000 | 0.958 | 0.000 | 0.001 |
|  |  | Blank | Mid | 0.040 | 1.000 | -0.001 | 0.001 |
|  |  |  | NIVD | 0.000 | 0.965 | 0.000 | 0.001 |
|  |  | Mid | NIVD | 0.000 | 0.995 | 0.000 | 0.001 |
|  | ***Group*** | ***Time (I)*** | ***Time (J)*** |  |  |  |  |
| AF | Normal | 1w | 2w | 0.015 | 1.000 | -0.001 | 0.001 |
|  |  |  | 4w | 0.015 | 1.000 | -0.001 | 0.001 |
|  |  | 2w | 4w | -0.006 | 1.000 | -0.001 | 0.001 |
|  | Blank | 1w | 2w | -0.014 | 1.000 | -0.001 | 0.001 |
|  |  |  | 4w | 0.009 | 1.000 | -0.001 | 0.001 |
|  |  | 2w | 4w | 0.023 | 1.000 | -0.001 | 0.001 |
|  | Mid | 1w | 2w | -0.002 | 0.002* | -0.003 | -0.001 |
|  |  |  | 4w | -0.004 | 0.000* | -0.005 | -0.003 |
|  |  | 2w | 4w | -0.002 | 0.003* | -0.003 | -0.001 |
|  | NIVD | 1w | 2w | -0.002 | 0.001* | -0.004 | -0.001 |
|  |  |  | 4w | -0.002 | 0.008* | -0.003 | 0.000 |
|  |  | 2w | 4w | 0.000 | 0.919 | -0.001 | 0.002 |
|  | ***Time*** | ***Group (I)*** | ***Group (J)*** |  |  |  |  |
|  | 1w | Normal | Blank | 0.024 | 1.000 | -0.002 | 0.002 |
|  |  |  | Mid | 0.011 | 1.000 | -0.002 | 0.002 |
|  |  |  | NIVD | -0.001 | 0.900 | -0.002 | 0.001 |
|  |  | Blank | Mid | -0.013 | 1.000 | -0.002 | 0.002 |
|  |  |  | NIVD | -0.001 | 0.873 | -0.002 | 0.001 |
|  |  | Mid | NIVD | -0.001 | 0.889 | -0.002 | 0.001 |
|  | 2w | Normal | Blank | -0.019 | 1.000 | -0.002 | 0.002 |
|  |  |  | Mid | -0.002 | 0.005* | -0.004 | 0.000 |
|  |  |  | NIVD | -0.003 | 0.000* | -0.004 | -0.001 |
|  |  | Blank | Mid | -0.002 | 0.005* | -0.004 | 0.000 |
|  |  |  | NIVD | -0.003 | 0.000* | -0.004 | -0.001 |
|  |  | Mid | NIVD | -0.001 | 0.775 | -0.002 | 0.001 |
|  | 4w | Normal | Blank | 0.018 | 1.000 | -0.002 | 0.002 |
|  |  |  | Mid | -0.004 | 0.000* | -0.006 | -0.002 |
|  |  |  | NIVD | -0.002 | 0.000* | -0.004 | -0.001 |
|  |  | Blank | Mid | -0.004 | 0.000* | -0.006 | -0.002 |
|  |  |  | NIVD | -0.002 | 0.000* | -0.004 | -0.001 |
|  |  | Mid | NIVD | 0.002 | 0.043* | 0.020 | 0.003 |
|  | ***Group*** | ***Time (I)*** | ***Time (J)*** |  |  |  |  |
| Homo-EP | Normal | 1w | 2w | -0.017 | 1.000 | -0.003 | 0.003 |
|  |  |  | 4w | 0.009 | 1.000 | -0.003 | 0.003 |
|  |  | 2w | 4w | 0.013 | 1.000 | -0.003 | 0.003 |
|  | Blank | 1w | 2w | 0.000 | 0.999 | -0.003 | 0.003 |
|  |  |  | 4w | -0.001 | 0.954 | -0.003 | 0.002 |
|  |  | 2w | 4w | 0.000 | 0.977 | -0.003 | 0.002 |
|  | Mid | 1w | 2w | 0.001 | 0.768 | -0.002 | 0.004 |
|  |  |  | 4w | 0.000 | 0.994 | -0.002 | 0.003 |
|  |  | 2w | 4w | -0.001 | 0.891 | -0.003 | 0.002 |
|  | NIVD | 1w | 2w | -0.002 | 0.144 | -0.005 | 0.000 |
|  |  |  | 4w | -0.003 | 0.034* | -0.005 | 0.000 |
|  |  | 2w | 4w | -0.001 | 0.915 | -0.003 | 0.002 |
|  | ***Time*** | ***Group (I)*** | ***Group (J)*** |  |  |  |  |
|  | 1w | Normal | Blank | 0.000 | 1.000 | -0.003 | 0.003 |
|  |  |  | Mid | -0.005 | 0.000* | -0.008 | -0.002 |
|  |  |  | NIVD | -0.003 | 0.019* | -0.006 | 0.000 |
|  |  | Blank | Mid | -0.005 | 0.000* | -0.008 | -0.002 |
|  |  |  | NIVD | -0.003 | 0.044* | -0.006 | -0.035 |
|  |  | Mid | NIVD | 0.002 | 0.455 | -0.001 | 0.005 |
|  | 2w | Normal | Blank | 0.000 | 0.999 | -0.003 | 0.003 |
|  |  |  | Mid | -0.004 | 0.001* | -0.007 | -0.001 |
|  |  |  | NIVD | -0.005 | 0.000* | -0.008 | -0.002 |
|  |  | Blank | Mid | -0.004 | 0.005* | -0.007 | -0.001 |
|  |  |  | NIVD | -0.005 | 0.000* | -0.008 | -0.002 |
|  |  | Mid | NIVD | -0.001 | 0.815 | -0.004 | 0.002 |
|  | 4w | Normal | Blank | -0.001 | 0.973 | -0.004 | 0.002 |
|  |  |  | Mid | -0.005 | 0.000* | -0.008 | -0.002 |
|  |  |  | NIVD | -0.006 | 0.000* | -0.009 | -0.003 |
|  |  | Blank | Mid | -0.004 | 0.002* | -0.007 | -0.001 |
|  |  |  | NIVD | -0.005 | 0.000* | -0.008 | -0.002 |
|  |  | Mid | NIVD | -0.001 | 0.847 | -0.004 | 0.002 |
|  | ***Group*** | ***Time (I)*** | ***Time (J)*** |  |  |  |  |
| Contra-EP | Normal | 1w | 2w | -0.019 | 1.000 | -0.004 | 0.004 |
|  |  |  | 4w | 0.008 | 1.000 | -0.004 | 0.004 |
|  |  | 2w | 4w | 0.013 | 1.000 | -0.004 | 0.004 |
|  | Blank | 1w | 2w | -0.015 | 1.000 | -0.004 | 0.004 |
|  |  |  | 4w | 0.007 | 1.000 | -0.004 | 0.004 |
|  |  | 2w | 4w | 0.016 | 1.000 | -0.004 | 0.004 |
|  | Mid | 1w | 2w | -0.002 | 0.406 | -0.007 | 0.002 |
|  |  |  | 4w | -0.006 | 0.003* | -0.010 | -0.002 |
|  |  | 2w | 4w | -0.003 | 0.141 | -0.008 | 0.001 |
|  | NIVD | 1w | 2w | -0.006 | 0.001* | -0.010 | -0.002 |
|  |  |  | 4w | -0.010 | 0.000* | -0.014 | -0.006 |
|  |  | 2w | 4w | -0.004 | 0.079 | -0.008 | 0.000 |
|  | ***Time*** | ***Group (I)*** | ***Group (J)*** |  |  |  |  |
|  | 1w | Normal | Blank | -0.019 | 1.000 | -0.005 | 0.005 |
|  |  |  | Mid | -0.001 | 0.999 | -0.005 | 0.004 |
|  |  |  | NIVD | -0.003 | 0.554 | -0.007 | 0.002 |
|  |  | Blank | Mid | -0.001 | 0.999 | -0.005 | 0.004 |
|  |  |  | NIVD | -0.003 | 0.557 | -0.007 | 0.002 |
|  |  | Mid | NIVD | -0.002 | 0.834 | -0.007 | 0.003 |
|  | 2w | Normal | Blank | -0.015 | 1.000 | -0.005 | 0.005 |
|  |  |  | Mid | -0.003 | 0.359 | -0.008 | 0.001 |
|  |  |  | NIVD | -0.009 | 0.000* | -0.013 | -0.004 |
|  |  | Blank | Mid | -0.003 | 0.368 | -0.008 | 0.001 |
|  |  |  | NIVD | -0.009 | 0.000* | -0.013 | -0.004 |
|  |  | Mid | NIVD | -0.006 | 0.007* | -0.010 | -0.001 |
|  | 4w | Normal | Blank | -0.012 | 1.000 | -0.005 | 0.005 |
|  |  |  | Mid | -0.007 | 0.001* | -0.011 | -0.002 |
|  |  |  | NIVD | -0.013 | 0.000* | -0.017 | -0.008 |
|  |  | Blank | Mid | -0.007 | 0.001* | -0.011 | -0.002 |
|  |  |  | NIVD | -0.013 | 0.000* | -0.017 | -0.008 |
|  |  | Mid | NIVD | -0.006 | 0.003* | -0.011 | -0.002 |
| *The mean difference is significant at the 0.05 level. | | | | | | | |
