# Supplementary material for: A novel rat model of vertebral inflammation–induced intervertebral disc degeneration mediated by activating cGAS/STING molecular pathway
Source: J Cell Mol Med. 2021 Sep 3;25(20):9567–85. doi: 10.1111/jcmm.16898 (PMC8505843; doi:10.1111/jcmm.16898)
Supplement: Supplementary file 6 — Table S5 [file JCMM-25-9567-s003.docx]

| **Table S5. Simple effect analysis for pairwise comparisons between group and time in IL-6 (IHC)** | | | | | | | |
| --- | --- | --- | --- | --- | --- | --- | --- |
| **Location** | **Subjects(AOD)** | | | **Mean Difference** | ***P*** | **95% Confidence Interval for Difference** | |
|  | ***Group*** | ***Time (I)*** | ***Time (J)*** | **(I－J)** |  | **Lower Bound** | **Upper Bound** |
| NP | Normal | 1w | 2w | 0.002 | 0.750 | -0.003 | 0.006 |
|  |  |  | 4w | 0.001 | 0.808 | -0.003 | 0.006 |
|  |  | 2w | 4w | 0.000 | 1.000 | -0.004 | 0.004 |
|  | Blank | 1w | 2w | 0.000 | 0.995 | -0.004 | 0.005 |
|  |  |  | 4w | -0.001 | 0.930 | -0.005 | 0.003 |
|  |  | 2w | 4w | -0.001 | 0.835 | -0.006 | 0.003 |
|  | Mid | 1w | 2w | 0.003 | 0.288 | -0.001 | 0.007 |
|  |  |  | 4w | 0.003 | 0.287 | -0.001 | 0.007 |
|  |  | 2w | 4w | 0.006 | 1.000 | -0.004 | 0.004 |
|  | NIVD | 1w | 2w | 0.001 | 0.973 | -0.004 | 0.005 |
|  |  |  | 4w | 0.002 | 0.458 | -0.002 | 0.007 |
|  |  | 2w | 4w | 0.002 | 0.720 | -0.003 | 0.006 |
|  | ***Time*** | ***Group (I)*** | ***Group (J)*** |  |  |  |  |
|  | 1w | Normal | Blank | 0.001 | 0.996 | -0.004 | 0.006 |
|  |  |  | Mid | -0.002 | 0.843 | -0.007 | 0.003 |
|  |  |  | NIVD | 0.000 | 1.000 | -0.005 | 0.004 |
|  |  | Blank | Mid | -0.003 | 0.479 | -0.007 | 0.002 |
|  |  |  | NIVD | -0.001 | 0.962 | -0.006 | 0.003 |
|  |  | Mid | NIVD | 0.001 | 0.956 | -0.003 | 0.006 |
|  | 2w | Normal | Blank | 0.000 | 1.000 | -0.005 | 0.004 |
|  |  |  | Mid | -0.001 | 0.999 | -0.005 | 0.004 |
|  |  |  | NIVD | -0.001 | 0.964 | -0.006 | 0.003 |
|  |  | Blank | Mid | 0.000 | 1.000 | -0.005 | 0.004 |
|  |  |  | NIVD | -0.001 | 0.989 | -0.006 | 0.004 |
|  |  | Mid | NIVD | -0.001 | 0.999 | -0.005 | 0.004 |
|  | 4w | Normal | Blank | -0.001 | 0.958 | -0.006 | 0.003 |
|  |  |  | Mid | -0.001 | 1.000 | -0.005 | 0.004 |
|  |  |  | NIVD | 0.000 | 1.000 | -0.004 | 0.005 |
|  |  | Blank | Mid | 0.001 | 0.996 | -0.004 | 0.006 |
|  |  |  | NIVD | 0.002 | 0.868 | -0.003 | 0.006 |
|  |  | Mid | NIVD | 0.001 | 0.995 | -0.004 | 0.006 |
|  | ***Group*** | ***Time (I)*** | ***Time (J)*** |  |  |  |  |
| AF | Normal | 1w | 2w | 0.008 | 1.000 | -0.011 | 0.011 |
|  |  |  | 4w | 0.035 | 1.000 | -0.011 | 0.011 |
|  |  | 2w | 4w | 0.033 | 1.000 | -0.011 | 0.011 |
|  | Blank | 1w | 2w | 0.000 | 1.000 | -0.012 | 0.011 |
|  |  |  | 4w | 0.000 | 1.000 | -0.011 | 0.011 |
|  |  | 2w | 4w | 0.000 | 1.000 | -0.011 | 0.011 |
|  | Mid | 1w | 2w | -0.039 | 0.000* | -0.050 | -0.027 |
|  |  |  | 4w | -0.044 | 0.000* | -0.056 | -0.033 |
|  |  | 2w | 4w | -0.006 | 0.537 | -0.017 | 0.006 |
|  | NIVD | 1w | 2w | -0.012 | 0.032* | -0.023 | -0.001 |
|  |  |  | 4w | -0.020 | 0.000* | -0.031 | -0.009 |
|  |  | 2w | 4w | -0.008 | 0.250 | -0.019 | 0.003 |
|  | ***Time*** | ***Group (I)*** | ***Group (J)*** |  |  |  |  |
|  | 1w | Normal | Blank | 0.000 | 1.000 | -0.012 | 0.013 |
|  |  |  | Mid | -0.001 | 1.000 | -0.013 | 0.012 |
|  |  |  | NIVD | -0.009 | 0.259 | -0.022 | 0.003 |
|  |  | Blank | Mid | -0.001 | 1.000 | -0.013 | 0.012 |
|  |  |  | NIVD | -0.009 | 0.241 | -0.022 | 0.003 |
|  |  | Mid | NIVD | -0.009 | 0.338 | -0.021 | 0.004 |
|  | 2w | Normal | Blank | 0.000 | 1.000 | -0.013 | 0.012 |
|  |  |  | Mid | -0.039 | 0.000* | -0.052 | -0.027 |
|  |  |  | NIVD | -0.021 | 0.000* | -0.034 | -0.009 |
|  |  | Blank | Mid | -0.039 | 0.000* | -0.052 | -0.027 |
|  |  |  | NIVD | -0.021 | 0.000* | -0.034 | -0.009 |
|  |  | Mid | NIVD | 0.018 | 0.001* | 0.005 | 0.030 |
|  | 4w | Normal | Blank | -0.037 | 1.000 | -0.013 | 0.012 |
|  |  |  | Mid | -0.045 | 0.000* | -0.057 | -0.033 |
|  |  |  | NIVD | -0.029 | 0.000* | -0.042 | -0.017 |
|  |  | Blank | Mid | -0.045 | 0.000* | -0.057 | -0.032 |
|  |  |  | NIVD | -0.029 | 0.000* | -0.042 | -0.017 |
|  |  | Mid | NIVD | 0.016 | 0.006* | 0.003 | 0.028 |
|  | ***Group*** | ***Time (I)*** | ***Time (J)*** |  |  |  |  |
| Homo-EP | Normal | 1w | 2w | -0.043 | 1.000 | -0.008 | 0.008 |
|  |  |  | 4w | -0.011 | 1.000 | -0.008 | 0.008 |
|  |  | 2w | 4w | 0.040 | 1.000 | -0.008 | 0.008 |
|  | Blank | 1w | 2w | 0.000 | 1.000 | -0.008 | 0.008 |
|  |  |  | 4w | -0.004 | 0.624 | -0.011 | 0.004 |
|  |  | 2w | 4w | -0.004 | 0.600 | -0.012 | 0.004 |
|  | Mid | 1w | 2w | -0.020 | 0.000* | -0.028 | -0.012 |
|  |  |  | 4w | -0.026 | 0.000* | -0.034 | -0.018 |
|  |  | 2w | 4w | -0.006 | 0.162 | -0.014 | 0.002 |
|  | NIVD | 1w | 2w | -0.009 | 0.019* | -0.017 | -0.001 |
|  |  |  | 4w | -0.012 | 0.001* | -0.020 | -0.004 |
|  |  | 2w | 4w | -0.003 | 0.726 | -0.011 | 0.005 |
|  | ***Time*** | ***Group (I)*** | ***Group (J)*** |  |  |  |  |
|  | 1w | Normal | Blank | -0.001 | 0.999 | -0.010 | 0.007 |
|  |  |  | Mid | -0.014 | 0.000* | -0.023 | -0.005 |
|  |  |  | NIVD | -0.028 | 0.000* | -0.037 | -0.019 |
|  |  | Blank | Mid | -0.013 | 0.001* | -0.022 | -0.004 |
|  |  |  | NIVD | -0.027 | 0.000* | -0.035 | -0.018 |
|  |  | Mid | NIVD | -0.014 | 0.000* | -0.023 | -0.005 |
|  | 2w | Normal | Blank | -0.001 | 1.000 | -0.010 | 0.008 |
|  |  |  | Mid | -0.034 | 0.000* | -0.043 | -0.025 |
|  |  |  | NIVD | -0.037 | 0.000* | -0.046 | -0.028 |
|  |  | Blank | Mid | -0.033 | 0.000* | -0.042 | -0.024 |
|  |  |  | NIVD | -0.036 | 0.000* | -0.045 | -0.027 |
|  |  | Mid | NIVD | -0.003 | 0.942 | -0.012 | 0.006 |
|  | 4w | Normal | Blank | -0.005 | 0.581 | -0.014 | 0.004 |
|  |  |  | Mid | -0.041 | 0.000* | -0.049 | -0.032 |
|  |  |  | NIVD | -0.040 | 0.000* | -0.049 | -0.031 |
|  |  | Blank | Mid | -0.036 | 0.000* | -0.044 | -0.027 |
|  |  |  | NIVD | -0.035 | 0.000* | -0.044 | -0.027 |
|  |  | Mid | NIVD | 0.000 | 1.000 | -0.008 | 0.009 |
|  | ***Group*** | ***Time (I)*** | ***Time (J)*** |  |  |  |  |
| Contra-EP | Normal | 1w | 2w | -0.016 | 1.000 | -0.007 | 0.007 |
|  |  |  | 4w | 0.013 | 1.000 | -0.007 | 0.007 |
|  |  | 2w | 4w | 0.029 | 1.000 | -0.007 | 0.007 |
|  | Blank | 1w | 2w | 0.011 | 1.000 | -0.007 | 0.007 |
|  |  |  | 4w | 0.000 | 1.000 | -0.007 | 0.007 |
|  |  | 2w | 4w | 0.000 | 1.000 | -0.007 | 0.007 |
|  | Mid | 1w | 2w | -0.006 | 0.075 | -0.013 | 0.000 |
|  |  |  | 4w | -0.032 | 0.000* | -0.038 | -0.025 |
|  |  | 2w | 4w | -0.025 | 0.000* | -0.032 | -0.019 |
|  | NIVD | 1w | 2w | -0.022 | 0.000* | -0.029 | -0.016 |
|  |  |  | 4w | -0.033 | 0.000* | -0.039 | -0.026 |
|  |  | 2w | 4w | -0.010 | 0.001* | -0.017 | -0.004 |
|  | ***Time*** | ***Group (I)*** | ***Group (J)*** |  |  |  |  |
|  | 1w | Normal | Blank | 0.000 | 1.000 | -0.008 | 0.007 |
|  |  |  | Mid | -0.001 | 1.000 | -0.008 | 0.007 |
|  |  |  | NIVD | -0.001 | 1.000 | -0.008 | 0.007 |
|  |  | Blank | Mid | -0.001 | 1.000 | -0.008 | 0.007 |
|  |  |  | NIVD | 0.000 | 1.000 | -0.008 | 0.007 |
|  |  | Mid | NIVD | 0.050 | 1.000 | -0.007 | 0.008 |
|  | 2w | Normal | Blank | -0.065 | 1.000 | -0.008 | 0.007 |
|  |  |  | Mid | -0.007 | 0.081 | -0.014 | 0.000 |
|  |  |  | NIVD | -0.023 | 0.000* | -0.030 | -0.015 |
|  |  | Blank | Mid | -0.007 | 0.088 | -0.014 | 0.001 |
|  |  |  | NIVD | -0.023 | 0.000* | -0.030 | -0.015 |
|  |  | Mid | NIVD | -0.016 | 0.000* | -0.023 | -0.008 |
|  | 4w | Normal | Blank | -0.012 | 1.000 | -0.007 | 0.007 |
|  |  |  | Mid | -0.032 | 0.000* | -0.040 | -0.025 |
|  |  |  | NIVD | -0.033 | 0.000* | -0.041 | -0.026 |
|  |  | Blank | Mid | -0.032 | 0.000* | -0.040 | -0.025 |
|  |  |  | NIVD | -0.033 | 0.000* | -0.041 | -0.026 |
|  |  | Mid | NIVD | -0.001 | 0.999 | -0.009 | 0.006 |
| *The mean difference is significant at the 0.05 level. | | | | | | | |
